# ESBL/*p*AmpC-Producing *Escherichia coli* Causing Urinary Tract Infections in Non-Related Companion Animals and Humans

**DOI:** 10.3390/antibiotics11050559

**Published:** 2022-04-22

**Authors:** Adriana Belas, Cátia Marques, Juliana Menezes, Luís Telo da Gama, Patrícia Cavaco-Silva, Constança Pomba

**Affiliations:** 1 Centre for Interdisciplinary Research in Animal Health (CIISA), Faculty of Veterinary Medicine, University of Lisbon, 1300-477 Lisbon, Portugal or adriana.belas@ulusofona.pt (A.B.); or catia.marques@ulusofona.pt (C.M.); julianamenezes@fmv.ulisboa.pt (J.M.); ltgama@fmv.ulisboa.pt (L.T.d.G.); 2 Associate Laboratory for Animal and Veterinary Sciences (AL4AnimalS), 1300-477 Lisbon, Portugal; 3 Faculty of Veterinary Medicine, Lusófona University, 1749-024 Lisbon, Portugal; 4 Centro de Investigação Interdisciplinar Egas Moniz, Instituto Universitário Egas Moniz, 2829-511 Caparica, Portugal; montezpat@gmail.com; 5 Technophage, 1649-028 Lisboa, Portugal

**Keywords:** *Escherichia coli*, ESBL/AmpC, pathogenicity, companion animals, humans, urinary tract infection

## Abstract

Urinary tract infections (UTI) caused by *Escherichia coli* are frequently diagnosed in humans and companion animals. Extended-spectrum beta-lactamase (ESBL)- and cephalosporinase (*p*AmpC)-producing *Escherichia coli* are worldwide-disseminated and frequently multidrug-resistant, hence leading to treatment failure and public health concerns. This study aimed to characterize and compare ESBL/*p*AmpC-producing *E. coli* strains causing community-acquired UTI in companion animals and non-related humans. Third-generation cephalosporin (3GC)-resistant *E. coli* (companion animals *n* = 35; humans *n* = 85) isolated from patients with UTI were tested against 14 antimicrobials following CLSI guidelines. PCR-based assays were used to detect the major *E. coli* phylogenetic groups, pathogenicity associated-islands (PAIs), virulence genes, and ESBLs/*p*AmpC resistance genes. ESBL/*p*AmpC-producing *E. coli* isolates were typed by multi-locus sequence typing (MLST) and PCR. *E. coli* strains from companion animals and humans shared two MDR high-risk clonal lineages: ST131 and ST648. To the best of our knowledge, this study reports the first description of *E. coli* ST131 clade C1-M27 and the clonal lineage ST131 clade A in humans with community-acquired UTI in Portugal. Considering that companion animals with UTI are generally treated at home by the owners, measures should be implemented to avoid the spread of multidrug-resistant high-risk clones to humans and their household environment.

## 1. Introduction

Urinary tract infections (UTI) may be caused by the uropathogenic *Escherichia coli* (UPEC), which is one of the extraintestinal pathogenic *E. coli* pathotypes (ExPEC), and one of the most frequent etiologic agents of UTI worldwide both in humans and companion animals [1,2,3,4].

The increase in antimicrobial resistance caused by the dissemination of resistant and multidrug-resistant (MDR) bacteria, such as extended-spectrum beta-lactamase (ESBL), cephalosporinase (AmpC) and carbapenemase-producing *E. coli*, is a current global threat responsible for thousands of deaths each year [5,6]. ESBL/AmpC and carbapenemase-producing *E. coli* are frequently MDR, thus causing treatment failure due to their ability to hydrolyze third- and fourth-generation cephalosporins or carbapenems, which have been considered critically important antimicrobials to human and veterinary medicine [5]. Moreover, in recent decades, some studies have alerted us to the emergence of MDR high-risk clonal lineages of clinically significant bacteria in companion animals, raising public-health concerns, since infected and colonized companion animals may contribute to the spread of such bacteria among humans, domestic animals, wildlife, and the environment [4,7,8,9]. The need for a global action plan to address the antimicrobial resistance crises requires a One Health approach supported by scientific data that can be used to raise awareness of decision-makers and the general population [6].

The production of beta-lactamase (ESBL/AmpC and carbapenemase) enzymes is an important factor that promotes the dissemination of high-risk clonal lineages that are also frequently MDR. The most prevalent of these are the ESBL CTX-M-like enzymes, although others, such as TEM and SHV enzymes and the plasmid-mediated AmpC (*p*AmpC) are also common. There is a large body of research showing that the distribution of ESBL/*p*AmpC varies geographically and according to the animal species considered. Interestingly, CTX-M-15 is one of the most frequent ESBLs detected among *E. coli* isolated from companion animals and humans in Europe and the Americas [10].

Besides the association with various antimicrobial resistance determinants, the classification as an international MDR high-risk clonal lineage is also linked with the bacterial pathogenicity, global distribution, ability to colonize and persist in the hosts for more than 6 months, capacity for transmission between hosts, and the ability to cause recurrent infections [11]. However, information about the pathogenicity and virulence of *E. coli* strains isolated from companion animals is still scarce.

The population structure of ESBL/*p*AmpC-producing *E. coli* is currently dominated globally by several high-risk clonal lineages, including the ST131, ST648, ST69, ST393, ST405, and ST410 lineages [11,12]. ST131 is one of the most successful *E. coli* clonal lineages, it is disseminated worldwide and is frequently a CTX-M-15 producer [8]. Although seemingly more frequent in humans, the ST131 lineage is also being increasingly detected in companion animals [8]. *E. coli* ST131 has been grouped into different clades, which are usually associated with specific *fimH* (type-1 fimbriae) alleles: clade A (*fimH*41 ST131-O16), clade B (*fimH*22 ST131-O25b), and clade C (*fimH*30 including ST131-O25b *fimH*30-R/*fimH*30-Rx). *E. coli* ST131 clade C1-M27 is associated with *bla*_CTX-M-27_, and C2 with *bla*_CTX-M-15_ [11,12]. The ST648 is also an important pandemic clonal lineage as it is frequently an ESBL producer and commonly causes infection in humans and companion animals [13]. The frequency and geographic distribution of beta-lactamase enzymes and *E. coli* clonal lineages may change over time, thus creating the need for continuous monitoring in animals and humans.

With the growing contact between companion animals and humans, the risk of animal-to-human transfer of ESBL/*p*AmpC-producing bacteria is a concern [14]. The risk of inter-species transfer of antimicrobial resistant bacteria is supported by previous studies that have demonstrated that companion animals may share UPEC with the remaining human household members [15,16,17,18,19]. Interestingly, humans may also be a reservoir of UPEC for their companion animals as seen with *E. coli* O25b:H4-B2-ST131. This clonal lineage has dramatically spread during recent decades and some of its clades, such as the *H30*Rx C2 (C2/*H30*Rx), have been linked to the dissemination of ESBLs, especially the CTX-M-15 enzyme [15,16,17,18,19]. The dissemination of ESBL/*p*AmpC-producing *E. coli* is a complex issue since their emergence, ecology, and association with virulence are still poorly understood both in human and companion animal infections. Thus, this study aimed to characterize and compare the antimicrobial resistance, virulence profile, phylogenetic distribution, and predominant clonal lineages of ESBL/*p*AmpC-producing *E. coli* strains causing UTI in companion animals and non-related humans.

## 2. Results

### 2.1. Antimicrobial Resistance and Phylogenetic Group of 3GC-Resistant E. coli

From a total of 330 non-duplicate *E. coli* isolates included from companion animals with UTI, 10.6% (*n* = 35/330) were resistant to third-generation cephalosporin (3GC). A high proportion of these 3GC-resistant *E. coli* were also resistant to ciprofloxacin (74.3%, *n* = 26/35), norfloxacin (71.4%, *n* = 25/35), trimethoprim/sulfamethoxazole (71.4%, *n* = 25/35), gentamicin (40.0%, *n* = 14/35), and tobramycin (31.4%, *n* = 11/35). Overall, 71.4% (*n* = 25/35) of these strains were MDR. However, no resistance to carbapenems was detected. Regarding 3GC-resistant *E. coli* from humans with UTI, these showed higher resistance frequencies against fluoroquinolones (88.2%, *n* = 75/85), trimethoprim/sulfamethoxazole (74.1%, *n* = 63/85), gentamicin (37.6%, *n* = 32/85), and tobramycin (49.4%, *n* = 42/85). Overall, 84.7% (*n* = 72/85) of the *E. coli* strains were MDR (Table 1).

Regarding the phylogenetic group, 3GC-resistant *E. coli* isolates from companion animals belonged mainly to group-D (48.6%, *n* = 17/35) and 3GC-resistant *E. coli* strains from humans belonged mainly to group-B2 (67.1%, *n* = 57/85) (Table 2).

The frequency of ESBL-producing *E. coli* was significantly higher in 3GC-resistant strains from humans with UTI than in companion animals (*p* < 0.0001). ESBLs were detected in *E. coli* belonging to all phylogenetic groups, including group-A which is considered less pathogenic. Nevertheless, group-B (including group-B1 and group-B2) predominated (Table 3).

*p*AmpC-producing *E. coli* strains belonged mainly to group-D in both groups, and were significantly more frequent in companion animals (94.1% in *E. coli* from companion animal; 40.0% in *E. coli* from humans, *p* < 0.0001) (Table 3).

Although *bla*_SHV_ was detected among ESBL-producing *E. coli*, *bla*_CTX-M-type_ ESBL clearly predominated in both groups. Several types of CTX-M enzymes were found showing high diversity of these ESBLs in UPEC, especially in strains from humans. Nevertheless, *bla*_CTX-M-15_ predominated in both groups (Table 4).

Regarding *p*AmpC, only *bla*_CMY-2_ was found, being the predominant antimicrobial resistance mechanisms responsible for 3GC-resistance in *E. coli* from companion animals. Interestingly, the cefoxitin resistance phenotype of four 3GC-resistant *E. coli* strains from companion animals could not be explained by any of the tested genes; thus, other mechanism of resistance were likely involved.

Moreover, carbapenemase genes were not detected in either 3GC-resistant *E. coli* collections, which is in line with the carbapenem-susceptible phenotype of these strains.

### 2.2. Pathogenicity Island Markers and Virulence Genotyping of 3GC-Resistant E. coli

Eight UPEC pathogenicity islands (PAIs) were screened in all 3GC-resistant *E. coli* strains. The most prevalent PAIs among strains from humans and companion animal were PAI_IV536_ (91.8%, *n* = 78/85; and 74.3%, *n* = 26/35, respectively), followed by PAI_CFT073_ (78.8%, *n* = 67/85; and 54.3%, *n* = 19/35, respectively) (Table 5).

All *E. coli* strains were positive for the *ecpA* gene, the major pilin subunit of *E. coli* common pilus, in both groups. Furthermore, the *papEF* operon segment, *iucD*, *hlyA*, and *cnf1* were also frequent in both groups (Table 6). However, the cytotoxic necrotizing factor-1 (*cnf1* gene) and aerobactin siderophore (*iucD* gene) frequencies were significantly higher in *E. coli* strains from humans (*p* = 0.012 and *p* = 0.0002, respectively) (Table 6).

### 2.3. Clonal Lineages of ESBL/pAmpC-Producing E. coli

The companion animal strains were distributed in 15 sequence types (STs), while the human strains belonged to 19 STs (Table 7). The most common STs among ESBL/*p*AmpC-producing *E. coli* strains from companion animals with UTI were the ST648 (*n* = 11), ST131 (*n* = 5), ST539 (*n* = 2), and ST1775 (*n* = 2) (Table 7).

Regarding the *E. coli* ST648 clonal lineage from companion animals, the PAI_IV536_-PAI_ICFT073_ (*n* = 5) combination was the most frequently detected. Different virulence gene profiles were found, but the most prevalent was *ecpA*-*papEF* (*n* = 7) (Table S1).

ST131 *E. coli* isolated from companion animals included two *E. coli* ST131 C2/*H30*Rx clades harboring the *bla*_CTX-M-15_ and one ST131C1/*H30*R1 (C1-non27) sub-clade harboring the *bla*_CTX-M-1_. The *E. coli* ST131 C1/*H30*R1 (C1-M27) clade harboring the *bla*_CTX-M-27_ was not detected in companion animals while the remaining ST131 *E. coli* strains remained unclassified by this assay (Table S1). For ST131 *E. coli* strains from companion animals, PAI_I536_-PAI_IIJ96_-PAI_ICFT073_-PAI_IV536_-PAI_IICFT073_ (*n* = 2) and PAI_ICFT073_-PAI_IV536_-PAI_IICFT073_ (*n* = 2), were the combinations detected most frequently. However, different profiles of virulence genes were also found (Table S1). Among phylogroup-B1, *E. coli* from companion animals belonged to ST539 (*n* = 2), one harboring *bla*_CMY-2_ and one harboring *bla*_CTX-M-1type_; followed by ST533 (*n* = 1, harboring *bla*_CTX-M-15_ and *bla*_CMY-2_) and ST224 (*n* = 1, harboring *bla*_CTX-M-32_). Regarding phylogroup-A *E. coli* strains from companion animals, these belonged to an unassigned ST (*n* = 1, harboring *bla*_CTX-M-15_), ST609 (*n* = 1, harboring *bla*_CTX-M-32_), ST88 (*n* = 1, harboring *bla*_CTX-M-1_), and ST23 (*n* = 1, harboring *bla*_CTX-M-32_) (Table 7).

The most common clonal lineages among ESBL/*p*AmpC-producing *E. coli* strains from humans were ST131 (*n* = 57) and ST453 (*n* = 6) (Table 7). As in companion animals, *E. coli* strains isolated from humans and from different phylogenetic groups also harbored diverse ESBLs/*p*AmpC genes: group-A (*n* = 4, *bla*_CTX-M-32_; *n* = 3, *bla*_CTX-M-1_; *n* = 1, *bla*_CTX-M-14_; *n* = 1, *bla*_CTX-M-27_; *n* = 1, *bla*_CTX-M-15_; and *n* = 1, *bla*_CTX-M-9like_); group-B1 (*n* = 1, *bla*_SHV-12_; *n* = 4, *bla*_CTX-M-14_; *n* = 4, *bla*_CTX-M-1_; *n* = 2, *bla*_CTX-M-15_; and *n* = 1, *bla*_CTX-M-2_); group-B2 (*n* = 42, *bla*_CTX-M-15_; *n* = 5, *bla*_CTX-M-27_; *n* = 3, *bla*_CTX-M-9like_; *n* = 2, *bla*_CTX-M-1_; *n* = 1, *bla*_CTX-M-14_; *n* = 1, *bla*_CTX-M-32_; and *n* = 3, *bla*_CMY-2_); and group-D (*n* = 2, *bla*_CTX-M-15_; *n* = 1, *bla*_CTX-M-14_; and *n* = 2, *bla*_CMY-2_) (Table 7). Among group-B2, *E. coli* strains from humans belonged mainly to the ST131-C2/*H30*Rx clade harboring the *bla*_CTX-M-15_ gene (71.9%, *n* = 41/57). The ST131-C1-M27 *E. coli* sub-clade harboring the *bla*_CTX-M-27_ gene was detected in 8.8% (*n* = 5/57) of ST131 strains from humans. The clonal lineage O16-H5-ST131 (clade A) was also detected (1.8%, *n* = 1/57) and found to harbor *bla*_CTX-9like_ ESBL. Finally, three strains isolated from humans belonging to the pandemic clone O25b:H4-B2-ST131 were found to be *p*AmpC-producers due to *bla*_CMY-2_ (Table S2). Regarding PAIs, the ST131 *E. coli* strains from humans were frequently positive for the following combinations: PAI_I536_-PAI_IIJ96_-PAI_II536_-PAI_ICFT073_-PAI_IV536_-PAI_IICFT073_ (*n* = 27) and PAI_ICFT073_-PAI_IV536_-PAI_IICFT073_ (*n* = 23). The distribution of the different virulence genes showed that the most prevalent profiles in strains from humans belonging to group-B2 were *ecpA*-*iucD* (*n* = 17) and *ecpA*-*papEF*-*sfaDE*-*hlyA*-*cnf1*-*iucD* (*n* = 15) (Table S2).

Notably, ESBL/*p*AmpC-producing *E. coli* from companion animals and humans with UTI belonged to two MDR high-risk clonal lineages, namely the ST131 and ST648. Moreover, the ST88 and ST354 clonal lineages were also shared by companion animals and humans (Table 7, Tables S1 and S2).

## 3. Discussion

This study showed that ESBL/*p*AmpC-producing *E. coli* from companion animals and humans with UTI may harbor a big diversity of clinically relevant beta-lactamases and were associated with several virulence determinants.

*E. coli* strains isolated from companion animals were frequently associated with the presence of *bla*_CTX-M-15_ and *bla*_CMY-2_ genes, while those isolated from humans were associated with *bla*_CTX-M-15_ and *bla*_CTX-M-1_. The high prevalence and disseminations of *bla*_CTX-M-15_ in *E. coli* isolated from animals and humans agrees with studies conducted worldwide [10]. Moreover, CMY-2-producing *E. coli* strains belonged mainly to the phylogenetic group-D, which is consistent with a previous study conducted in the United States [20].

Despite the similarities between the *E. coli* strains isolated from both study groups with respect to mobile genetic determinants of antimicrobial resistance and virulence, the phylogenetic group-B2 and group-D was significantly more common in humans and companion animals, respectively. This finding may point to different *E. coli* host species adaptations contributing both to the global dissemination of overlapping antimicrobial resistance and virulence determinants.

The *E. coli* phylogenetic group-A, which is usually considered commensal and less pathogenic, was found to cause UTI in both companion animals and humans. Furthermore, this phylogroup was associated with a high diversity of globally disseminated *bla*_CTX-M_ genes, such as *bla*_CTX-M-15_ and *bla*_CTX-M-32_. These findings highlight the high dissemination efficiency of plasmid-mediated beta-lactamases that may lead to therapeutic failure even in infections caused by less pathogenic strains.

Overall, 3GC-resistant *E. coli* strains belonging to the phylogenetic group-B2 had the higher number of PAI markers. This association of the group-B2 with several PAI markers is in line with previous reports of UPEC strains [21]. Interestingly, the most frequent PAI combination pattern was related to strains containing PAI_IV536_ and PAI_ICFT073_. These PAI markers contain fimbrial adhesins and iron-uptake-system encoding genes that seem to be important for UPEC fitness and effective host colonization of the urinary tract. A high prevalence of fimbrial adhesin-encoding genes (such as, *papEF* operon segment) has been described in *E. coli* isolated from human patients diagnosed with UTI, thus highlighting the importance of these structures in the pathogenesis of UTI [22]. Furthermore, PAI_ICFT073_ also carries the toxin hemolysin A (*hlyA* gene), that is responsible for the creation of pores in the host cell membranes leading to cell lysis [22]. Notably, PAI_IV536_ and PAI_ICFT073_ were detected in 74.3% and 54.3% of *E. coli* strains from companion animals, respectively. Strains isolated from humans had an even higher prevalence of these PAIs (91.8% and 78.8%, respectively).

The uropathogenic-specific protein gene (*usp*) was detected only in one *E. coli* ST131 strain, which was isolated from a dog diagnosed with UTI in 2015. This protein is a genotoxin active against mammalian cells that can induce characteristics of apoptosis and has been associated with *E. coli* isolates from pyelonephritis, prostatitis, and bacteremia of urinary tract origin. It has been proposed that the *usp* gene provides immunity to its producer and enhances infectivity of the urinary tract [23,24].

Regarding the *E. coli* population structure, four sequence types were detected in companion animals and humans—the ST131 and ST648 MDR high-risk clonal lineages, and the ST88 and ST354 clonal lineages. The fact that the *E. coli* strains included in this study, from companion animals and humans, were collected in different years and from non-related patients is considered a study limitation, since it could have limited the detection of additional *E. coli* STs that are able to cause UTI in both groups. It should be noted that the results from this study may differ from studies including samples from a more recent timeframe as consequence of natural evolution and dissemination of beta-lactamases and *E. coli* clonal lineages. Nevertheless, the retrospective nature of this study is important as it contributes to the global understanding of the ecology of this common pathogen. Furthermore, this study includes data about virulence, which is still seldomly studied in strains from companion animals.

The ST131 clonal lineage harboring *bla*_CTX-M-15_ or *bla*_CTX-M-14_ has been detected in *E. coli* strains isolated from companion animals in many other countries [17,19,25]. However, the *E. coli* O25b:H4-ST131 harboring the *bla*_CMY-2_ gene has been rarely described [26,27]. In Japan, between 2005 and 2010, the *bla*_CTX-M-14_ gene was found to be the most common, followed by *bla*_CTX-M-15_ and *bla*_CTX-M-2_ [25]. In the present study, *bla*_CTX-M-14_ was also only detected in *E. coli* strains isolated from humans. The *bla*_CTX-M-27_ gene, a single-nucleotide variant of *bla*_CTX-M-14_, is being increasingly detected among *E. coli* strains isolated from humans and companion animals with UTI in the United States, Asia, and Europe [25,28,29,30]. In this study, the *bla*_CTX-M-27_ gene was only detected in one strain of human origin. To the best of our knowledge, this is the first description of the ST131 C1/*H30*R1 *E. coli* subclade C1-M27 and *bla*_CMY-2_-producing *E. coli* O25b:H4-ST131 in humans with community-acquired UTI from Portugal. Some reports have documented an increase in the number of the ST131 C1/H30R1 *E. coli* clade C1-M27 since the late 2000s. Isolates of this emerging clade have been reported in clinical samples from humans of Japan, France, Germany, Berlin, Geneva, Madrid, and Utrecht [25,31,32,33]; and also in companion animals, birds and urban seagulls [30,34,35]. Moreover, in northern Portugal, the C1-M27 clade has been isolated from fecal samples of healthy humans [36]. It is noteworthy that the ST131 C1/H30R1 *E. coli* clade C1-M27 has been shown to have a higher dissemination rate than the O25b:H4-ST131-H30Rx [37,38]. This dissemination rate may be accelerated by the expression of advantageous virulence determinants directed to the intestinal tract colonization, which is of particular importance to patients receiving antimicrobials or frequently admitted to hospital settings [33]. Thus, human colonization by clade C1-M27 should be monitored to improve preventive measures against infection and colonization of companion animals.

Previous studies about clinical *E. coli* strains showed that the O16-H5-ST131 clonal lineage (clade A) is globally distributed [39]. In this study, an *E. coli* O16:H5-ST131 harboring *bla*_CTX-9-like_ was detected, which, to the best of our knowledge, is the first description in a human with community-acquired UTI from Portugal. The lower frequency of the O16-B2-ST131 in this study when compared to other countries [39,40,41], may reflect geographical and temporal differences in its distribution. Furthermore, according to Matsumura et al. [40], the O16-B2-ST131 clonal lineage has a lower prevalence of resistance to fluoroquinolones and ceftriaxone than O25b-B2-ST131 isolates [39,40,41]. This may also explain the low detection of O16-B2-ST131 in this study since it was focused on 3GC-resistant *E. coli*. Considering its high frequency in humans, the development of rapid molecular detection methods to identify ST131, while waiting for culture and antimicrobial susceptibility, could aid in a more effective initial antibiotic therapy and the reduction of its dissemination [39].

The *E. coli* ST648 high-risk clonal lineage is pandemic and globally reported in healthy and diseased humans and companion animals worldwide [13,20,42,43,44,45,46,47,48]. *E. coli* ST648 isolated from human infection may harbor several ESBL/*p*AmpC and carbapenemases, contributing to its dissemination [13,42,49]. As in ST131, the *E. coli* ST648 is frequently associated with CTX-M enzymes, namely CTX-M-15 [13]. The 3GC-resistant *E. coli* ST648 strains from this study, were all found to be CMY-2-producers. Furthermore, the CMY-2-producing ST648 clonal lineage was very common among isolates from companion animals, which is in line with previous studies [20,43]. Being a clonal lineage that is high-risk to humans, monitoring of ST648 in companion animals is of the upmost importance.

Interestingly, the *E. coli* ST88 and ST354 clonal lineages have been associated with poultry and broiler meat, suggesting that farm animals may be reservoirs of *E. coli* that are able to cause extraintestinal diseases in humans and companion animals [26,50,51,52,53,54]. *E. coli* ST354 has also been isolated from clinical samples of companion animals from Australia, and has been suggested to have a propensity to persist and circulate in animal-care facilities [55]. To the best of our knowledge, this is the first report of a CMY-2-producer *E. coli* ST354 in companion animals with UTI in Europe.

*E. coli* ST10 and ST410, other two important pandemic clonal lineages, were found in humans with UTI from this study [11,56]. *E. coli* ST410 seems to be a successful ExPEC clonal lineage like ST131 [57,58]. *E. coli* ST410 was first described, in 2016, in China [59] and since then has been reported worldwide in humans, companion animals, wildlife, and the environment [58,60,61,62,63]. Notably, in 2017, the ST410 was detected in companion animals with UTI from China [45]. Nevertheless, only a few studies have detected this clonal lineage in *E. coli* isolated from companion animals [45,62,63]. The *E. coli* ST10 clonal lineage has been reported in samples from animals (birds, swine, and sheep), in healthy human feces, and in humans with UTI. This clonal lineage is also usually associated with several 3GC-resistance genes such as *bla*_CTX-M-14_ and *bla*_CTX-M-15_ [64,65,66,67]. However, in the present study, the ST10 clone was associated with *bla*_CTX−M−1_. Although not detected in this study, ST410 has been previously described in isolates from companion animals, again, highlighting their possible role in the dissemination of high-risk clonal lineages.

The results from this study have high clinical relevance since it is shown that 3GC-resistant *E. coli* strains causing UTI in companion animals not only may belong to MDR high-risk clonal lineages, but are also likely to harbor critically important mobile genetic determinants associated with high pathogenicity or antimicrobial resistance.

## 4. Materials and Methods

### 4.1. Bacterial Isolates

Three hundred and thirty non-duplicate uropathogenic *E. coli* (UPEC) were isolated, between 1999 to 2015, from companion animals with UTI at the Laboratory of Antibiotic Resistance, the Faculty of Veterinary Medicine, University of Lisbon, Portugal. Furthermore, 85 non-duplicate 3GC-resistant *E. coli* isolates from humans with community-acquired UTI were obtained in 2013 from a diagnostic laboratory in the Lisbon area. 3GC-resistance was determined by antimicrobial susceptibility testing using cefotaxime or ceftazidime as surrogates, as described in Section 4.2.

Identification and confirmation of the isolate species was performed by a previously described PCR targeting the *E. coli gadA* gene [68].

### 4.2. Antimicrobial Susceptibility Testing

Antimicrobial susceptibility testing was carried by the disk-diffusion method according to Clinical Laboratory Standards Institute (CLSI) guidelines [69,70]. The antimicrobial agents tested were: amoxicillin 25 μg or ampicillin 10 μg, amoxicillin/clavulanate acid 30 μg, cefotaxime 30 μg, ceftazidime 30 μg, cefoxitin 30 μg, imipenem 10 μg, meropenem 10 μg, gentamicin 10 μg, tobramycin 10 μg, amikacin 30 μg, ciprofloxacin 5 μg, enrofloxacin 5 μg, norfloxacin 10 μg, nitrofurantoin 300 μg, and trimethoprim/sulfamethoxazole 25 μg. ESBL production was confirmed in all 3GC-resistant isolates by the double-disk synergy test and the results were interpreted according to the to CLSI guidelines. *E. coli* ATCC (American Type Culture Collection) 25,922 was used as a reference strain for antimicrobial susceptibility quality control.

Antimicrobial categories were used to characterize multidrug resistance as previously proposed by Magiorakos et al. [71].

### 4.3. Molecular Detection of Antimicrobial Resistance Genes

DNA extraction was conducted using a boiling method [72]. Antimicrobial resistance genes were investigated in resistant and intermediate resistant strains.

3GC-resistant *E. coli* were screened for *bla*_CTX-M-type_ genes by PCR [73]. Positive isolates for *bla*_CTX-M-type_ were further tested by PCR for *bla*_CTX-M-group1_, *bla*_CTX-M-group2_, and *bla*_CTX-M-group9_ [74] and positive amplicons were submitted to nucleotide sequencing. Cefoxitin-resistant *E. coli* isolates were tested using a multiplex-PCR with specific primers targeting plasmid-borne genes encoding AmpC β-lactamases (*bla*_CIT_, *bla*_LAT_, *bla*_ACT_, *bla*_MIR_, *bla*_FOX_, *bla*_MOX_, and *bla*_DHA_), as previously described [75]. Positive samples for the group CIT were submitted to nucleotide sequencing after PCR amplification targeting the entire *bla*_CMY-2_ gene [76]. 3GC-resistant *E. coli* negative for *bla*_CTX-M-type_ or AmpC genes were tested for the presence of *bla*_TEM-type_ and *bla*_SHV-type_ ESBL genes [72].

Strains were screened by PCR for the presence of common carbapenemase genes (*bla*_IMP_, *bla*_OXA_, *bla*_VIM_, *bla*_NDM_, and *bla*_KPC_), as previously described [77].

### 4.4. Uropathogenic Escherichia coli Phylogenetic Typing, Pathogenicity Island Markers, and Virulence Genotyping

Phylogenetic typing was performed in all 3GC-resistant *E. coli* strains to determine the main phylogenetic groups (A, B1, B2, and D) according to the amplification of *chuA* gene, *yjaA* gene, and *TspE4C2* fragment [78].

Eight UPEC PAIs were screened by multiplex PCR assays, as previously described [28,29]. PCR reactions were split in three separate multiplex assays: multiplex A for PAI_III536_, PAI_IV536_, and PAI_IICFT073_; multiplex B1 for PAI_IIJ96_ and PAI_I536_; and multiplex B2 for PAI_II536_, PAI_IJ96_, and PAI_ICFT073_. Negative and positive controls (*E. coli* CFT073, *E. coli* 536, and *E. coli* J96) were used in all PCRs [21,79].

3GC-resistant *E. coli* strains were screened by PCR for the presence of several virulence determinants: mediate adhesion (p-fimbrial adhesion genes *papEF* operon segment), Sfa fimbrial and Afa afimbrial adhesins (*sfa* and *afa* genes, respectively), toxin (α-hemolysin *hlyA* gene from the alpha-hemolysin operon), cytotoxic necrotizing factor 1 (*cnf-1* gene), aerobactin siderophore (*iucD* gene) [80], the major pilin subunit of *E. coli* common pilus (*ecpA* gene), and the bacteriocin-like genotoxin uropathogenic specific protein (*usp* gene) [9]. Negative and positive controls (*E. coli* CFT073, *E. coli* 536, *E. coli* J96, and *E. coli* KS52) were used in all PCRs.

### 4.5. ESBL/pAmpC-Producing Escherichia coli Multi-Locus Sequence Typing

The ST131 clonal lineage, O16/O25b types, and the ST131-*H30*Rx clade were identified, as previously described by PCR [39,81,82].

*E. coli* strains not belonging to the ST131 clonal lineage were typed by MLST. Briefly, the seven housekeeping genes of the *E. coli* MLST scheme (*adk*, *fumC*, *gyrB*, *icd*, *mdh*, *purA*, and *recA*) were amplified by PCR using the primers and amplification conditions previously described in https://enterobase.warwick.ac.uk/ (accessed on 10 February 2020) [83]. PCR products were purified using the NZYTech Gel Pure Kit (NZYTech-Genes and Enzymes, Lisbon, Portugal) and sequencing was performed by Stabvida (Caparica, Portugal). Sequence quality was confirmed using Ugene Unipro software (Unipro, Novosibirsk, Russia) and the respective alleles and sequence types were retrieved using the publicly available *E. coli* MLST database.

### 4.6. Statistical Analysis

The SAS statistical software package for Windows v. 9.4 (SAS Institute Inc., Cary, NC, USA) was used for statistical analysis. The Fisher’s exact test was used for comparisons between groups (two by two analysis of contingency tables) with a *p* value of 0.05.

## 5. Conclusions

This study showed that 3GC-resistant *E. coli* from companion animals and humans with community-acquired UTI frequently belong to important pandemic high-risk clonal lineages and harbor clinically relevant antimicrobial resistance and virulence determinants that are easily disseminated. Considering the close contact between companion animals and humans in modern society, the dissemination of pandemic *E. coli* clones, such as ST131-C2/*H30*Rx (*bla*_CTXM-15_) and ST648, in patients with UTI requires the joint action of human and veterinary medicine. Although the degree of inter-species transmission and zoonotic/zooanthroponotic potential of such bacteria is complex to evaluate, the high frequency of common PAI markers and CTX-M enzymes reported in this study, highlights that the link between humans and companion animals goes beyond sharing specific *E. coli* clones. As appraised by the scientific community, a One Health approach is required to fully grasp the dissemination dynamics of such bacterial clones and/or their antimicrobial resistance and virulence mobile genetic determinants.

## Figures and Tables

**Table 1:** Antimicrobial resistance of 3GC-resistant *E. coli* isolated from companion animals and humans with UTI.

Antimicrobials	Companion Animal (*N* = 35) ^a^ %R (*n*)	Human-CA (*N* = 85) ^a^ %R (*n*)	*p* Value *
Ampicillin/amoxicillin	100% (*n* = 35)	100% (*n* = 85)	N.s.
Amoxicillin/clavulanate	77.1% (*n* = 27)	27.1% (*n* = 23)	<0.0001
Cefoxitin	62.9% (*n* = 22)	8.2% (*n* = 7)	<0.0001
Cefotaxime	91.4% (*n* = 32)	100% (*n* = 85)	0.023
Ceftazidime	62.8% (*n* = 22)	42.4% (*n* = 36)	0.047
Imipenem	0.0% (*n* = 0)	0.0% (*n* = 0)	N.s.
Meropenem	0.0% (*n* = 0)	0.0% (*n* = 0)	N.s.
Ciprofloxacin	74.3% (*n* = 26)	88.2% (*n* = 75)	0.096
Norfloxacin	71.4% (*n* = 25)	88.2% (*n* = 75)	0.033
Nitrofurantoin	5.7% (*n* = 2)	2.4% (*n* = 2)	N.s.
Gentamicin	40.0% (*n* = 14)	37.6% (*n* = 32)	0.838
Amikacin	5.7% (*n* = 2)	10.6% (*n* = 9)	0.506
Tobramycin	31.4% (*n* = 11)	49.4% (*n* = 42)	0.105
Trimethoprim/sulfamethoxazole	71.4% (*n* = 25)	74.1% (*n* = 63)	0.822
Multidrug resistant	71.4% (*n* = 25)	84.7% (*n* = 72)	0.125

%R, percentage of resistant strains; Human-CA, human community-acquired UTI; *n*, number of strains; ^a^ the number shown (*N*) is the total number of strains tested. * *p* value < 0.05 was considered statistically significant. N.s. not significant to calculate.

**Table 2:** Phylogenetic groups of 3GC-resistant *E. coli* isolated from companion animals and humans with UTI.

Phylogenetic Group	Companion Animal (*N* = 35) ^a^ % (*n*)	Human-CA (*N* = 85) ^a^ % (*n*)	*p* Value *
Group A	22.9% (*n* = 8)	12.9% (*n* = 11)	0.187
Group B1	11.4% (*n* = 4)	14.1% (*n* = 12)	0.777
Group B2	17.1% (*n* = 6)	67.0% (*n* = 57)	<0.0001
Group D	48.6% (*n* = 17)	5.9% (*n* = 5)	<0.0001

Human-CA, human community-acquired UTI; *n*, number of strains; ^a^ the number shown (*N*) is the total number of strains tested. * *p* value < 0.05 was considered statistically significant.

**Table 3:** ESBL and *p*AmpC-producing *E. coli* by phylogenetic group.

Phylogenetic Group	Companion Animal (*N* = 35) ^a^		Phylogenetic Group	Human-CA (*N* = 85) ^a^		*p* Value * ESBL Genes	*p* Value * pAMPc Genes
	ESBL Genes % (*n*)	*p*AmpC Genes % (*n*)		ESBL Genes % (*n*)	*p*AmpC Genes % (*n*)		
A (*n* = 8)	62.5% (*n* = 5)	0.0% (*n* = 0)	A (*n* = 11)	100% (*n* = 11)	0.0% (*n* = 0)	0.057	N.s
B1 (*n* = 4)	75.0% (*n* = 3)	50.0% (*n* = 2)	B1 (*n* = 12)	100% (*n* = 12)	0.0% (*n* = 0)	0.250	0.050
B2 (*n* = 6)	83.3% (*n* = 5)	33.3% (*n* = 2)	B2 (*n* = 57)	94.7% (*n* = 54)	5.3% (*n* = 3)	0.337	0.067
D (*n* = 17)	5.8% (*n* = 1)	94.1% (*n* = 16)	D (*n* = 5)	60.0% (*n* = 3)	40.0% (*n* = 2)	0.024	<0.0001
Overall	40.0% (*n* = 14)	57.1% (*n* = 20)	Overall	94.1% (*n* = 80)	5.9% (*n* = 5)	<0.0001	<0.0001

% percentage of strains; Human-CA, human community-acquired UTI; *n*, number of strains; ^a^ the number shown (*N*) is the total number of strains tested; ESBL, extended-spectrum β-lactamase; *p*AmpC, *p*AmpC β-lactamases. * *p* value < 0.05 was considered statistically significant. N.s. not significant to calculate.

**Table 4:** ESBLs and *p*AmpC genes in 3GC-ressistant *E. coli* isolated from companion animals and humans with UTI.

Beta-Lactamase Gene	Companion Animal (*N* = 35) ^a^ % (*n*)	Human-CA (*N* = 85) ^a^ % (*n*)
*bla*_SHV-12_	0.0% (*n* = 0)	1.2% (*n* = 1)
*bla*_CTX-M-1_	5.7% (*n* = 2)	10.6% (*n* = 9)
*bla*_CTX-M-1-type_	5.7% (*n* = 2)	0.0% (*n* = 0)
*bla*_CTX-M-15_	20.0% (*n* = 7)	54.2% (*n* = 46)
*bla*_CTX-M-15-type_	0.0% (*n* = 0)	1.2% (*n* = 1)
*bla*_CTX-M-32_	8.6% (*n* = 3)	5.9% (*n* = 5)
*bla*_CTX-M-9_	2.9% (*n* = 1)	0.0% (*n* = 0)
*bla*_CTX-M-9-type_	0.0% (*n* = 0)	4.7% (*n* = 4)
*bla*_CTX-M-14_	0.0% (*n* = 0)	8.2% (*n* = 7)
*bla*_CTX-M-27_	0.0% (*n* = 0)	7.1% (*n* = 6)
*bla*_CTX-M-2group_	0.0% (*n* = 0)	1.2% (*n* = 1)
*bla*_CMY-2_	57.1% (*n* = 20)	5.9% (*n* = 5)

% percentage of strains; Human-CA, human community-acquired UTI; *n*, number of strains; ^a^ the number (*N*) shown is the total number of strains tested.

**Table 5:** Frequency of pathogenicity island markers (PAIs) among 3GC-resistant *E. coli* strains from companion animals and humans with UTI.

Detected PAIs	Companion Animal (*N* = 35) ^a^ % (*n*)	Human-CA (*N* = 85) ^a^ % (*n*)	*p* Value *
PAI_IJ96_	0.0% (*n* = 0)	0.0% (*n* = 0)	N.s.
PAI_IIJ96_	11.4% (*n* = 4)	41.2% (*n* = 35)	0.004
PAI_I536_	8.6% (*n* = 3)	40.0% (*n* = 34)	0.0005
PAI_II536_	22.9% (*n* = 8)	35.3% (*n* = 30)	0.203
PAI_III536_	0.0% (*n* = 0)	0.0% (*n* = 0)	N.s.
PAI_IV536_	74.3% (*n* = 26)	91.8% (*n* = 78)	0.017
PAI_ICFT073_	54.3% (*n* = 19)	78.8% (*n* = 67)	0.013
PAI_IICFT073_	20.0% (*n* = 7)	69.4% (*n* = 59)	<0.0001

*n*, number of strains; Human-CA, human community-acquired UTI; ^a^ the number shown (*N*) is the total number of strains tested. * *p* value < 0.05 was considered statistically significant. N.s. not significant to calculate.

**Table 6:** Frequency of virulence genes among 3GC-resistant *E. coli* isolated from companion animals and humans with UTI.

Target Virulence Determinant	Target Gene	Companion Animal (*N* = 35) ^a^	Human-CA (*N* = 85) ^a^	*p* Value *
Pap fimbriae	*papEF* operon segment	45.7% (*n* = 16)	49.4% (*n* = 42)	0.841
Sfa fimbriae	*sfa*	20.0% (*n* = 7)	20.0% (*n* = 17)	N.s.
Afa afimbrial adhesin	*afa*	2.9% (*n* = 1)	9.4% (*n* = 8)	0.281
Alpha-hemolysin operon	*hlyA*	40.0% (*n* = 14)	42.4% (*n* = 36)	0.841
Cytotoxic necrotizing factor-1	*cnf1*	17.1% (*n* = 6)	41.2% (*n* = 35)	0.012
Aerobactin siderophore	*iucD*	48.6% (*n* = 17)	83.5% (*n* = 71)	0.0002
*E. coli* common pilus	*ecpA*	100% (*n* = 35)	100% (*n* = 85)	N.s.
Uropathogenic specific protein	*usp*	2.9% (*n* = 1)	0.0% (*n* = 0)	0.292

*n*, number of strains; ^a^ the number shown (*N*) is the total number of strains tested; Human-CA, human community-acquired UTI. * *p* value < 0.05 was considered statistically significant. N.s. not significant to calculate.

**Table 7:** Sequence types of ESBLs/*p*AmpC-producing *E. coli* isolated from companion animals and humans with UTI.

Phylogroup	Sequence Type	Clonal Complex	β-Lactamase (ESBL/*p*AmpC)	Species (*n*)
A	ST10	10	*bla*_CTX-M-1_	Human (2)
A	ST23	23	*bla*_CTX-M-32_	Cat (1)
A	ST88	23	*bla*_CTX-M-1_	Dog (1)
			*bla*_CTX-M-15_	Human (1)
			*bla*_CTX-M-32_	Human (1)
A	ST90	23	*bla*_CTX-M-9like_	Human (1)
			*bla*_CTX-M-27_	Human (1)
A	ST167	10	*bla*_CTX-M-32_	Human (1)
A	ST540	-	*bla*_CTX-M-32_	Human (1)
A	ST609	46	*bla*_CTX-M-32_	Dog (1)
A	ST617	10	*bla*_CTX-M-1_	Human (1)
A	ST5257	-	*bla*_CTX-M-32_	Human (1)
A	ST6023	-	*bla*_CTX-M-14_	Human (1)
A	Unassigned ST *	-	*bla*_CTX-M-15_	Dog (1)
B1	ST58	155	*bla*_CTX-M-1_	Human (2)
B1	ST224	-	*bla*_CTX-M-32_	Cat (1)
B1	ST453	86	*bla*_SHV-12_	Human (1)
			*bla*_CTX-M-1_	Human (1)
			*bla*_CTX-M-15_	Human (1)
			*bla*_CTX-M-14_	Human (3)
B1	ST533	-	*bla*_CTX-M-15_ + *bla*_CMY-2_	Dog (1)
B1	ST539		*bla*_CTX-M-1like_	Dog (1)
			*bla*_CMY-2_	Cat (1)
B1	ST847	-	*bla*_CTX-M-14_	Human (1)
B1	ST1196	-	*bla*_CTX-M-1_	Human (1)
B1	ST1725	-	*bla*_CTX-M-15_	Human (1)
B1	ND	-	*bla*_CTX-M-2group_	Human (1)
B2	ST131	131	*bla*_CTX-M-1_	Cat (1), human (2)
			*bla*_CTX-M-15_	Dog (2), cat (1), human (42)
			*bla*_CTX-M-32_	Human (1)
			*bla*_CTX-M-9like_	Human (3)
			*bla*_CTX-M-14_	Human (1)
			*bla*_CTX-M-27_	Human (5)
			*bla*_CMY-2_	Dog (1), human (3)
B2	ST372	-	*bla*_CTX-M-15_	Dog (1)
D	ST57	350	*bla*_CMY-2_	Dog (1)
D	ST117	-	*bla*_CTX-M-15_	Human (1)
D	ST354	354	*bla*_CTX-M-14_	Human (1)
			*bla*_CMY-2_	Dog (1)
D	ST405	405	*bla*_CMY-2_	Dog (1)
D	ST410	-	*bla*_CTX-M-15_	Human (1)
D	ST648	648	*bla*_CTX-M-9_ + *bla*_CMY-2_	Cat (1)
			*bla*_CMY-2_	Dog (3), cat (7), human (1)
D	ST778	38	*bla*_CMY-2_	Human (1)
D	ST1775	-	*bla*_CMY-2_	Dog (2)
D	ST3258	-	*bla*_CMY-2_	Cat (1)

ND, not done; -, not applicable; *n*, number of strains; * new ST allelic profile [4].

## Data Availability

Data sharing is not applicable to this article.

## Supplementary Materials

The following supporting information can be downloaded at: https://www.mdpi.com/article/10.3390/antibiotics11050559/s1, Table S1: Genotypic characteristics of ESBLs/*p*AmpC-producing *E. coli* strains from companion animals with UTI (*N* = 31) from 1999–2015; Table S2: Genotypic characteristics of ESBLs/*p*AmpC-producing *E. coli* strains from humans with UTI (*N* = 85) from 2013.

## References

[1] Kaper J.B., Nataro J.P., Mobley H.L. (2004). Pathogenic *Escherichia coli*. Nat. Rev. Microbiol..

[2] Foxman B. (2010). The epidemiology of urinary tract infection. Nat. Rev. Urol..

[3] Jakobsen L., Spangholm D.J., Pedersen K., Jensen L.B., Emborg H.D., Agersø Y., Aarestrup F.M., Hammerum A.M., Frimodt-Møller N. (2010). Broiler chikens, broiler chiken meat, pigs and pork as sources of ExPEC related virulence genes and resistance in *Escherichia coli* isolates from community—Dwelling humans and UTI patients. Int. J. Food Microbiol..

[4] Marques C., Belas A., Franco A., Aboim C., Gama L.T., Pomba C. (2018). Increase in antimicrobial resistance and emergence of major international high-risk clonal lineages in dogs and cats with urinary tract infection: 16-year retrospective study. J. Antimicrob. Chemother..

[5] World Health Organization (WHO) (2019). Critically Important Antimicrobials for Human Medicine. 6th Revision WHO Advisory Group on Integrated Surveillance of Antimicrobial Resistance (AGISAR). https://apps.who.int/iris/bitstream/handle/10665/312266/9789241515528-eng.pdf.

[6] Ruiz J. (2021). Antimicrobial Resistance, from Bench-to-Publicside. Microbes Infect. Chemother..

[7] Ewers C., Antão E.M., Diehl I., Philipp H.C., Wieler L.H. (2009). Intestine and environment of the chicken as reservoirs for extraintestinal pathogenic *Escherichia coli* strains with zoonotic potential. Appl. Environ. Microbiol..

[8] Ewers C., Grobbel M., Stamm I., Kopp P.A., Diehl I., Semmler T., Fruth A., Beutlich J., Guerra B., Wieler L.H. (2010). Emergence of human pandemic O25:H4-ST131 CTX-M-15 extended-spectrum-beta-lactamase-producing *Escherichia coli* among companion animals. J. Antimicrob. Chemother..

[9] Narciso A., Nunes F., Amores T., Lito L., Melo-Cristino J., Duarte A. (2012). Persistence of uropathogenic *Escherichia coli* strains in the host for long periods of time: Relationship between phylogenetic groups and virulence factors. Eur. J. Clin. Microbiol. Infect. Dis..

[10] Ewers C., Bethe A., Semmler T., Guenther S., Wieler L.H. (2012). Extended-spectrum β-lactamase-producing and AmpC-producing *Escherichia coli* from livestock and companion animals, and their putative impact on public health: A global perspective. Clin. Microbiol. Infect..

[11] Mathers A.J., Peirano G., Pitout J.D. (2015). The role of epidemic resistance plasmids and international high-risk clones in the spread of multidrug-resistant Enterobacteriaceae. Clin. Microbiol. Rev..

[12] Peirano G., Pitout J.D.D. (2019). Extended-Spectrum β-Lactamase-Producing Enterobacteriaceae: Update on molecular epidemiology and treatment options. Drugs.

[13] Ewers C., Bethe A., Stamm I., Grobbel M., Kopp P.A., Guerra B., Stubbe M., Doi Y., Zong Z., Kola A. (2014). CTX-M-15-D-ST648 *Escherichia coli* from companion animals and horses: Another pandemic clone combining multiresistance and extraintestinal virulence?. J. Antimicrob. Chemother..

[14] Pomba C., Rantala M., Greko C., Baptiste K.E., Catry B., van Duijkeren E., Mateus A., Moreno M.A., Pyörälä S., Ružauskas M. (2017). Public health risk of antimicrobial resistance transfer from companion animals. J. Antimicrob. Chemother..

[15] Johnson J.R., Miller S., Johnston B., Clabots C., DebRoy C. (2009). Sharing of *Escherichia coli* Sequence Type ST131 and other multidrug-resistant and urovirulent *E. coli* strains among dogs and cats within a household. J. Clin. Microbiol..

[16] Johnson J.R., Menard M., Johnston B., Kuskowski M.A., Nichol K., Zhanel G. (2009). Epidemic clonal groups of *Escherichia coli* as a cause of antimicrobial—Resistant urinary tract infections in Canada, 2002–2004. Antimicrob. Agents Chemother..

[17] Pomba C., López-Cerero L., Bellido M., Serrano L., Belas A., Couto N., Cavaco-Silva P., Rodríguez-Baño J., Pascual A. (2014). Within-lineage variability of ST131 *Escherichia coli* isolates from humans and companion animals in the south of Europe. J. Antimicrob. Chemother..

[18] Barroso M., López-Cerero I., Navarro L., Gutiérrez-Gutiérrez M.D., Pascual B., Rodríguez-Baño J. (2018). Intestinal colonization due to *Escherichia coli* ST131: Risk factors and prevalence. Antimicrob. Resist. Infect. Control.

[19] Belas A., Marques C., Aboim C., Pomba C. (2019). Emergence of *Escherichia coli* ST131 *H30*/*H30*-Rx subclones in companion animals. J. Antimicrob. Chemother..

[20] Liu X., Thungrat K., Boothe D.M. (2016). Occurrence of OXA-48 carbapenemases and other β-lactamase genes in ESBL-producing multidrug resistant *Escherichia coli* from dogs and cats in the United States, 2009–2013. Front. Microbiol..

[21] Sabaté M., Moreno E., Perez T., Andreu A., Prats G. (2006). Pathogenicity Island markers in commensal and uropathogenic *Escherichia coli* isolates. Clin. Microbiol. Infect..

[22] Sarowska J., Futoma-Koloch B., Jama-Kmiecik A., Frej-Madrzak M., Ksiazczyk M., Bugla-Ploskonska G., Choroszy-Krol I. (2019). Virulence factors, prevalence and potential transmission of extraintestinal pathogenic *Escherichia coli* isolated from different sources: Recent reports. Gut Pathog..

[23] Nipič D., Podlesek Z., Budič M., Črnigoj M., Žgur-Bertok D. (2013). *Escherichia coli* uropathogenic-specific protein, Usp, is a bacteriocin-like genotoxin. J. Infect. Dis..

[24] Crnigoj M., Podlesek Z., Budič M., Zgur-Bertok D. (2014). The *Escherichia coli* uropathogenic-specific-protein-associated immunity protein 3 (Imu3) has nucleic acid -binding activity. BMC Microbiol..

[25] Matsumura Y., Pitout J.D., Gomi R., Matsuda T., Noguchi T., Yamamoto M., Peirano G., DeVinney R., Bradford P.A., Motyl M.R. (2016). Global *Escherichia coli* sequence Type 131 clade with *bla*_CTX-M-27_ gene. Emerg. Infect. Dis..

[26] Day M.J., Rodríguez I., van Essen-Zandbergen A., Dierikx C., Kadlec K., Schink A.K., Wu G., Chattaway M.A., DoNascimento V., Wain J. (2016). Diversity of STs, plasmids and ESBL genes among *Escherichia coli* from humans, animals and food in Germany, the Netherlands and the UK. J. Antimicrob. Chemother..

[27] Hansen K.H., Bortolaia V., Nielsen C.A., Nielsen J.B., Schønning K., Agersø Y., Guardabassi L. (2016). Host-specific patterns of genetic diversity among IncI1-Igamma and IncK plasmids encoding CMY-2 beta-lactamase in *Escherichia coli* isolates from humans, poultry meat, poultry, and dogs in Denmark. Appl. Environ. Microbiol..

[28] Harada K., Nakai Y., Kataoka Y. (2012). Mechanisms of resistance to cephalosporin and emergence of O25b-ST131 clone harboring CTX-M-27 β-lactamase in extraintestinal pathogenic *Escherichia coli* from dogs and cats in Japan. Microbiol. Immunol..

[29] Bevan E.R., Jones A.M., Hawkey P.M. (2017). Global epidemiology of CTX-M β-lactamases: Temporal and geographical shifts in genotype. J. Antimicrob. Chemother..

[30] Melo L.C., Haenni M., Saras E., Duprilot M., Nicolas-Chanoine M.H., Madec J.Y. (2019). Emergence of the C1-M27 cluster in ST131 *Escherichia coli* from companion animals in France. J. Antimicrob. Chemother..

[31] Birgy A., Bidet P., Levy C., Sobral E., Cohen R., Bonacorsi S. (2017). CTX-M-27-producing *Escherichia coli* of sequence type 131 and clade C1-M27, France. Emerg. Infect. Dis..

[32] Ghosh H., Doijad S., Falgenhauer L., Fritzenwanker M., Imirzalioglu C., Chakraborty T. (2017). *bla*_CTX-M-27_-encoding *Escherichia coli* sequence type 131 lineage C1-M27 clone in clinical isolates, Germany. Emerg. Infect. Dis..

[33] Merino I., Hernández-García M., Turrientes M.C., Pérez-Viso B., López-Fresneña N., Diaz-Agero C., Maechler F., Fankhauser-Rodriguez C., Kola A., Schrenzel J. (2018). Emergence of ESBL-producing *Escherichia coli* ST131-C1-M27 clade colonizing patients in Europe. J. Antimicrob. Chemother..

[34] Zendri F., Maciuca I.E., Moon S., Jones P.H., Wattret A., Jenkins R., Baxter A., Timofte D. (2020). Occurrence of ESBL-producing *Escherichia coli* ST131, including the H30-Rx and C1-M27 subclones, among urban seagulls from the United Kingdom. Microb. Drug Resist..

[35] Duggett N., Ellington M.J., Hopkins K.L., Ellaby N., Randall L., Lemma F., Teale C., Anjum M.F. (2021). Detection in livestock of the human pandemic *Escherichia coli* ST131 *fimH30*(R) clone carrying *bla*_CTX-M-27_. J. Antimicrob. Chemother..

[36] Rodrigues C., Machado E., Fernandes S., Peixe L., Novais Â. (2016). An update on faecal carriage of ESBL-producing Enterobacteriaceae by Portuguese healthy humans: Detection of the H30 subclone of B2-ST131 *Escherichia coli* producing CTX-M-27. J. Antimicrob. Chemother..

[37] Adler A., Gniadkowski M., Baraniak A., Izdebski R., Fiett J., Hryniewicz W., Malhotra-Kumar S., Goossens H., Lammens C., Lerman Y. (2012). Transmission dynamics of ESBL-producing *Escherichia coli* clones in rehabilitation wards at a tertiary care centre. Clin. Microbiol. Infect..

[38] Kurittu P., Khakipoor B., Jalava J., Karhukorpi J., Heikinheimo A. (2022). Whole-genome sequencing of extended-spectrum beta-lactamase-producing *Escherichia coli* from human infections in Finland revealed isolates belonging to internationally successful ST131-C1-M27 subclade but distinct from non-human sources. Front. Microbiol..

[39] Johnson J.R., Clermont O., Johnston B., Clabots C., Tchesnokova V., Sokurenko E., Junka A.F., Maczynska B., Denamur E. (2014). Rapid and specific detection, molecular epidemiology, and experimental virulence of the O16 subgroup within *Escherichia coli* sequence type 131. J. Clin. Microbiol..

[40] Matsumura Y., Yamamoto M., Nagao M., Ito Y., Takakura S., Ichiyama S., Kyoto-Shiga Clinical Microbiology Study Group (2013). Association of fluoroquinolone resistance, virulence genes, and IncF plasmids with extended-spectrum-β-lactamase-producing *Escherichia coli* sequence type 131 (ST131) and ST405 clonal groups. Antimicrob. Agents Chemother..

[41] Banerjee R., Johnson J.R. (2014). A new clone sweeps clean: The enigmatic emergence of *Escherichia coli* sequence type 131. Antimicrob. Agents Chemother..

[42] Huber H., Zweifel C., Wittenbrink M.M., Stephan R. (2013). ESBL-producing uropathogenic *Escherichia coli* isolated from dogs and cats in Switzerland. Vet. Microbiol..

[43] Tamang M.D., Nam H.M., Jang G.C., Kim S.R., Chae M.H., Jung S.C., Byun J.W., Park Y.H., Lim S.K. (2012). Molecular characterization of extended-spectrum-β-lactamase-producing and plasmid-mediated AmpC β-lactamase-producing *Escherichia coli* isolated from stray dogs in South Korea. Antimicrob. Agents Chemother..

[44] Toleman M.A., Bugert J.J., Nizam S.A. (2015). Extensively drug-resistant New Delhi metallo-β-lactamase-encoding bacteria in the environment, Dhaka, Bangladesh, 2012. Emerg. Infect. Dis..

[45] Li S., Liu J., Zhou Y., Miao Z. (2017). Characterization of ESBL-producing *Escherichia coli* recovered from companion dogs in Taiwan, China. J. Infect. Dev. Ctries..

[46] Solgi H., Giske C.G., Badmasti F., Aghamohammad S., Havaei S.A., Sabeti S., Mostafavizadeh K., Shahcheraghi F. (2017). Emergence of carbapenem resistant *Escherichia coli* isolates producing *bla*_NDM_ and *bla*_OXA-48_-like carried on IncA/C and IncL/M plasmids at two Iranian university hospitals. Infect. Genet. Evol..

[47] Sellera F.P., Fernandes M.R., Ruiz R., Falleiros A.C.M., Rodrigues F.P., Cerdeira L., Lincopan N. (2018). Identification of KPC-2-producing *Escherichia coli* in a companion animal: A new challenge for veterinary clinicians. J. Antimicrob. Chemother..

[48] Fernandes M.R., Sellera F.P., Moura Q., Gaspar V.C., Cerdeira L., Lincopan N. (2018). International high-risk clonal lineages of CTX-M-producing *Escherichia coli* F-ST648 in free-roaming cats, South America. Infect. Genet. Evol..

[49] Poirel L., Madec J.Y., Lupo A., Schink A.K., Kieffer N., Nordmann P., Schwarz S. (2018). Antimicrobial resistance in *Escherichia coli*. Microbiol. Spectr..

[50] Van Hoek A.H.A.M., Veenman C., Florijn A., Huijbers P.M.C., Graat E.A.M., de Greeff S., Dierikx C.M., van Duijkeren E. (2018). Longitudinal study of ESBL *Escherichia coli* carriage on an organic broiler farm. J. Antimicrob. Chemother..

[51] Borges C.A., Tarlton N.J., Riley L.W. (2019). *Escherichia coli* from commercial broiler and backyard chickens share sequence types, antimicrobial resistance profiles, and resistance genes with human extraintestinal pathogenic *Escherichia coli*. Foodborne Pathog. Dis..

[52] Wang M., Jiang M., Wang Z., Chen R., Zhuge X., Dai J. (2021). Characterization of antimicrobial resistance in chicken-source phylogroup F *Escherichia coli*: Similar populations and resistance spectrums between *E. coli* recovered from chicken colibacillosis tissues and retail raw meats in Eastern China. Poult. Sci..

[53] Mora A., Blanco M., López C., Mamani R., Blanco J.E., Alonso M.P., García-Garrote F., Dahbi G., Herrera A., Fernández A. (2011). Emergence of clonal groups O1:HNM-D-ST59, O15:H1-D-ST393, O20:H34/HNM-D-ST354, O25b:H4-B2-ST131 and ONT:H21,42-B1-ST101 among CTX-M-14-producing *Escherichia coli* clinical isolates in Galicia, northwest Spain. Int. J. Antimicrob. Agents.

[54] Ingram P.R., Rogers B.A., Sidjabat H.E., Gibson J.S., Inglis T.J.J. (2013). Co-selection may explain high rates of ciprofloxacin non-susceptible *Escherichia coli* from retail poultry reared without prior fluoroquinolone exposure. J. Med. Microbiol..

[55] Vangchhia B., Abraham S., Bell J.M., Collignon P., Gibson J.S., Ingram P.R., Johnson J.R., Kennedy K., Trott D.J., Turnidge J.D. (2016). Phylogenetic diversity, antimicrobial susceptibility and virulence characteristics of phylogroup F *Escherichia coli* in Australia. Microbiology.

[56] Campos A.C.C., Andrade N.L., Ferdous M., Chlebowicz M.A., Santos C.C., Correal J.C.D., Lo Ten Foe J.R., Rosa A.C.P., Damasco P.V., Friedrich A.W. (2020). Corrigendum: Comprehensive molecular characterization of *Escherichia coli* isolates from urine samples of hospitalized patients in Rio de Janeiro, Brazil. Front. Microbiol..

[57] Falgenhauer L., Imirzalioglu C., Ghosh H., Gwozdzinski K., Schmiedel J., Gentil K., Bauerfeind R., Kämpfer P., Seifert H., Michael G.B. (2016). Circulation of clonal populations of fluoroquinolone-resistant CTX-M-15-producing *Escherichia coli* ST410 in humans and animals in Germany. Int. J. Antimicrob. Agents.

[58] Schaufler K., Semmler T., Wieler L.H., Wöhrmann M., Baddam R., Ahmed N., Müller K., Kola A., Fruth A., Ewers C. (2016). Clonal spread and interspecies transmission of clinically relevant ESBL-producing *Escherichia coli* of ST410—Another successful pandemic clone?. FEMS Microbiol. Ecol..

[59] Qin S., Zhou M., Zhang Q., Tao H., Ye Y., Chen H., Xu L., Xu H., Wang P., Feng X. (2016). First identification of NDM-4-producing *Escherichia coli* ST410 in China. Emerg. Microbes Infect..

[60] Roer L., Overballe-Petersen S., Hansen F., Schønning K., Wang M., Røder B.L., Hansen D.S., Justesen U.S., Andersen L.P., Fulgsang-Damgaard D. (2018). *Escherichia coli* sequence type 410 is causing new international high-risk clones. mSphere.

[61] Falgenhauer L., Waezsada S.E., Gwozdzinski K., Ghosh H., Doijad S., Bunk B., Spröer C., Imirzalioglu C., Seifert H., Irrgang A. (2016). Chromosomal locations of mcr-1 and *bla*_CTX-M-15_ in fluoroquinolone-resistant *Escherichia coli* ST410. Emerg. Infect. Dis..

[62] Nigg A., Brilhante M., Dazio V., Clément M., Collaud A., Gobeli Brawand S., Willi B., Endimiani A., Schuller S., Perreten V. (2019). Shedding of OXA-181 carbapenemase-producing *Escherichia coli* from companion animals after hospitalisation in Switzerland: An outbreak in 2018. Eurosurveillance.

[63] Brilhante M., Menezes J., Belas A., Feudi C., Schwarz S., Pomba C., Perreten V. (2020). OXA-181-producing extraintestinal pathogenic *Escherichia coli* sequence type 410 isolated from a dog in Portugal. Antimicrob. Agents Chemother..

[64] Valverde A., Cantón R., Garcillán-Barcia M.P., Novais A., Galán J.C., Alvarado A., de la Cruz F., Baquero F., Coque T.M. (2009). Spread of *bla*(CTX-M-14) is driven mainly by IncK plasmids disseminated among *Escherichia coli* phylogroups A, B1, and D in Spain. Antimicrob. Agents Chemother..

[65] Bado I., Gutiérrez C., García-Fulgueiras V., Cordeiro N.F., Araújo Pirez L., Seija V., Bazet C., Rieppi G., Vignoli R. (2016). CTX-M-15 in combination with *aac(6′)-Ib-cr* is the most prevalent mechanism of resistance both in *Escherichia coli* and *Klebsiella pneumoniae*, including *K. pneumoniae* ST258, in an ICU in Uruguay. J. Glob. Antimicrob. Resist..

[66] Maluta R.P., Logue C.M., Casas M.R., Meng T., Guastalli E.A., Rojas T.C., Montelli A.C., Sadatsune T., de Carvalho Ramos M., Nolan L.K. (2014). Overlapped sequence types (STs) and serogroups of avian pathogenic (APEC) and human extra-intestinal pathogenic (ExPEC) *Escherichia coli* isolated in Brazil. PLoS ONE.

[67] Umpiérrez A., Bado I., Oliver M., Acquistapace S., Etcheverría A., Padola N.L., Vignoli R., Zunino P. (2017). Zoonotic potential and antibiotic resistance of *Escherichia coli* in neonatal calves in Uruguay. Microbes Environ..

[68] McDaniels A.E., Rice E.W., Reyes A.L., Johnson C.H., Haugland R.A., Stelma G.N. (1996). Confirmational identification of *Escherichia coli*, a comparison of genotypic and phenotypic assays for glutamate decarboxylase and beta-d-glucuronidase. Appl. Environ. Microbiol..

[69] Clinical and Laboratory Standards Institute (CLSI) (2019). Performance Standards for Antimicrobial Susceptibility Testing—27th Edition. Approved Standard. M100-S29.

[70] Clinical and Laboratory Standards Institute (CLSI) (2020). Performance Standards for Antimicrobial Disk and Dilution Susceptibility Tests for Bacteria Isolated from Animals—5th Edition Approved Standard.

[71] Magiorakos A.P., Srinivasan A., Carey R.B., Carmeli Y., Falagas M.E., Giske C.G., Harbarth S., Hindler J.F., Kahlmeter G., Olsson-Liljequist B. (2012). Multidrug-resistant, extensively drug-resistant and pandrug-resistant bacteria: An international expert proposal for interim standard definitions for acquired resistance. Clin. Microbiol. Infect..

[72] Pomba C., Mendonça N., Costa M., Louro D., Baptista B., Ferreira M., Correia J.D., Caniça M. (2006). Improved multiplex PCR method for the rapid detection of beta-lactamase genes in *Escherichia coli* of animal origin. Diagn. Microbiol. Infect. Dis..

[73] Edelstein M., Pimkin M., Palagin I., Edelstein I., Stratchounski L. (2003). Prevalence and molecular epidemiology of CTX-M Extended-Spectrumβ-Lactamase-Producing *Escherichia coli* and *Klebsiella pneumoniae* in Russian Hospitals. Antimicrob. Agents Chemother..

[74] Woodford N., Fagan E.J., Ellington M.J. (2006). Multiplex PCR for rapid detection of genes encoding CTX-M extended-spectrum (beta)-lactamases. J. Antimicrob. Chemother..

[75] Pérez F., Hanson N. (2002). Detection of plasmid-mediated AmpC beta-lactamase genes in clinical isolates by using Multiplex PCR. J. Clin. Microbiol..

[76] Belas A., Salazar A.S., Gama L.T., Couto N., Pomba C. (2014). Risk factors for faecal colonisation with *Escherichia coli* producing extended-spectrum and plasmid-mediated AmpC β-lactamases in dogs. Vet. Rec..

[77] Poirel L., Walsh T.R., Cuvillier V., Nordmann P. (2011). Multiplex PCR for detection of acquired carbapenemase genes. Diagn. Microbiol. Infect. Dis..

[78] Doumith M., Day M.J., Hope R., Wain J., Woodford N. (2012). Improved multiplex PCR strategy for rapid assignment of the four major *Escherichia coli* phylogenetic groups. J. Clin. Microbiol..

[79] Bronowski C., Smith S.L., Yokota K., Corkill J.E., Martin H.M., Campbell B.J., Rhodes J.M., Hart C.A., Winstanley C. (2008). A subset of mucosa associated *Escherichia coli* isolates from patients with colon cancer, but not Crohn’s disease, share pathogenicity islands with urinary pathogenic *E. coli*. Microbiology.

[80] Féria C.P., Correia J.C., Gonçalves J., Machado J. (2002). Detection of virulence factors in uropathogenic *Escherichia coli* isolated from humans, dogs and cats in Portugal. Adv. Exp. Med. Biol..

[81] Banerjee R., Robicsek A., Kuskowski M.A., Porter S., Johnston B.D., Sokurenko E., Tchesnokova V., Price L.B., Johnson J.R. (2013). Molecular epidemiology of *Escherichia coli* Sequence Type 131 and its *H30* and *H30*-Rx subclones among Extended-Spectrum-β-Lactamase-positive and -negative *E. coli* clinical isolates from the Chicago region, 2007 to 2010. Antimicrob. Agents Chemother..

[82] Colpan A., Johnston B., Porter S., Clabots C., Anway R., Thao L., Kuskowski M.A., Tchesnokova V., Sokurenko E.V., Johnson J.R. (2013). *Escherichia coli* sequence type 131 (ST131) subclone *H30* as an emergent multidrug-resistant pathogen among US veterans. Clin. Infect. Dis..

[83] Wirth T., Falush D., Lan R., Colles F., Mensa P., Wieler L.H., Karch H., Reeves P.R., Maiden M.C., Ochman H. (2006). Sex and virulence in *Escherichia coli*: An evolutionary perspective. Mol. Microbiol..

