# Supplementary material for: ESBL/pAmpC-Producing Escherichia coli Causing Urinary Tract Infections in Non-Related Companion Animals and Humans
Source: Antibiotics (Basel). 2022 Apr 22;11(5):559. doi: 10.3390/antibiotics11050559 (PMC9137695; doi:10.3390/antibiotics11050559)
Supplement: Supplementary file 1 [file antibiotics-11-00559-s001.zip › antibiotics-1682973-supplementary.pdf]

Table S1: Genotypic characteristics of ESBLs/*p*AmpC-producing *E. coli* strains from companion animals with UTI (*N*= 31) from 1999-2015

Strain	Year	Companion animal	Phylogenetic group	Clonal group	CC	Antimicrobial resistance[a]	ESBL and/or *p*AmpC genes	Pathogenicity island markers (PAIs)	Virulence genes
FMV434/00	2000	Dog	D	ST1775	-	AMP-AMC-CTX-CAZ-FOX-SXT	$bla_{\text{CMY-2}}$	PAI_{ICFT073}-PAI_{IV536}	*ecpA-iucD*
FMV457/00	2000	Dog	D	ST1775	-	AMP-AMC-CTX-CAZ-FOX-SXT	$bla_{\text{CMY-2}}$	PAI_{ICFT073}-PAI_{IV536}	*ecpA-iucD*
FMV1953/01	2001	Dog	D	ST57	CC350	AMP-AMC-CTX-CAZ-FOX-SXT-TOB-AK	$bla_{\text{CMY-2}}$	PAI_{ICFT073}-PAI_{IV536}	*ecpA-iucD*
FMV203/03	2003	Dog	D	ST405	CC405	AML-AMC-CTX-CIP-NOR-ENR	$bla_{\text{CMY-2}}$	PAI_{II536}-PAI_{IV536}	*ecpA – hlyA-cnf1*
FMV5825/04	2004	Dog	B2	O25b:H4-ST131-H30Rx	CC131	AMP-AMC-CTX-CAZ-FOX-SXT-CIP-NOR-ENR-CN-AK-TOB	$bla_{\text{CTX-M-15}}$	PAI_{II536}-PAI_{IIJ96}-PAI_{ICFT073}-PAI_{IV536}-PAI_{IICFT073}	*ecpA-hlyA-cnf1-sfaDE-papEF-iucD*
FMV6346/05	2005	Cat	B1	ST539	-	AMP-AMC-CTX-CAZ-FOX-CIP-NOR-ENR-CN	$bla_{\text{CMY-2}}$	none	*ecpA*
FMV3389/06	2006	Dog	D	ST354	CC354	AMP-AMC-CTX-CAZ-FOX-SXT-CIP-NOR-ENR-CN-TOB	$bla_{\text{CMY-2}}$	PAI_{II536}-PAI_{ICFT073}-PAI_{IV536}	*ecpA- hlyA-iucD*
FMV521/07	2007	Cat	B1	ST224	-	AMP-CTX-CAZ-CIP-NOR-ENR	$bla_{\text{CTX-M-32}}$	none	*ecpA*
FMV1630/07	2007	Dog	A	unassigned ST[b]	CC23	AMP-CTX-CAZ-SXT-CIP-NOR-ENR	$bla_{\text{CTX-M-15}}$	PAI_{IV536}	*ecpA*
FMV7261/07	2007	Dog	A	ST609	CC46	AMP-CTX-SXT-CIP-NOR-ENR	$bla_{\text{CTX-M-32}}$	PAI_{IV536}	*ecpA*
FMV635/08	2008	Cat	A	ST23	CC23	AMP-AMC-CTX-CAZ-SXT-CIP-NOR-ENR	$bla_{\text{CTX-M-32}}$	none	*ecpA*
FMV2777/08	2008	Cat	B2	O25b:H4-ST131-H30Rx	CC131	AMP-AMC-CTX-CAZ-CIP-NOR-ENR-CN-TOB	$bla_{\text{CTX-M-15}}$	PAI_{ICFT073}-PAI_{IV536}-PAI_{IICFT073}	*ecpA-hlyA-papEF-iucD*

FMV1952/10	2010	Cat	D	ST648	CC648	AMP-AMC-CTX-CAZ-FOX-SXT-CIP-NOR-ENR-CN-TOB	*bla*$_{\text{CTX-M-9}}$ + *bla*$_{\text{CMY-2}}$	PAI$_{\text{II536}}$-PAI$_{\text{IV536}}$	*ecpA-papEF*
FMV25/11	2011	Cat	D	ST648	CC648	AMP-AMC-CTX-CAZ-FOX-SXT-CIP-NOR-ENR-CN	*bla*$_{\text{CMY-2}}$	PAI$_{\text{ICFT073}}$-PAI$_{\text{IV536}}$	*ecpA-papEF*
FMV29/11	2011	Dog	D	ST648	CC648	AMP-AMC-CTX-CAZ-FOX-CIP-NOR-ENR-CN-AK	*bla*$_{\text{CMY-2}}$	PAI$_{\text{ICFT073}}$-PAI$_{\text{IV536}}$	*ecpA-papEF*
FMV469/13	2013	Dog	D	ST648	CC648	AMP-AMC-CTX-CAZ-FOX-SXT-CIP-NOR-ENR-CN-TOB	*bla*$_{\text{CMY-2}}$	PAI$_{\text{II536}}$-PAI$_{\text{IV536}}$	*ecpA-papEF*
FMV1389/13	2013	Cat	D	ST648	CC648	AML-AMC-CTX-FOX-SXT-CIP-NOR-ENR-CN	*bla*$_{\text{CMY-2}}$	PAI$_{\text{II536}}$-PAI$_{\text{ICFT073}}$-PAI$_{\text{IV536}}$	*ecpA-papEF*
FMV55/13	2013	Cat	D	ST648	CC648	AMP-AMC-CTX-FOX-SXT-ENR-NOR-CN	*bla*$_{\text{CMY-2}}$	PAI$_{\text{ICFT073}}$-PAI$_{\text{IV536}}$-PAI$_{\text{IICFT073}}$	*ecpA-sfaDE-papEF-iucD*
FMV58/13	2013	Cat	B2	O25b:H4-ST131-H30R1	CC131	AMP-CTX-SXT-CIP-NOR-ENR	*bla*$_{\text{CTX-M-1}}$	PAI$_{\text{ICFT073}}$-PAI$_{\text{IV536}}$-PAI$_{\text{IICFT073}}$	*ecpA- hlyA-sfaDE-papEF-iucD*
FMV4479/13	2013	Dog	B1	ST533	-	AMP-AMC-CTX-CAZ-FOX-SXT-CIP-NOR-ENR-TOB	*bla*$_{\text{CTX-M-15}}$ + *bla*$_{\text{CMY-2}}$	none	*ecpA*
FMV5338/13	2013	Dog	B2	O25b:H4-ST131-H30Rx	CC131	AMP-AMC-CTX-CIP-NOR-CN-TOB	*bla*$_{\text{CTX-M-15}}$	PAI$_{\text{I536}}$-PAI$_{\text{IIJ96}}$-PAI$_{\text{ICFT073}}$-PAI$_{\text{IV536}}$-PAI$_{\text{IICFT073}}$	*ecpA-hlyA, cnf1-afaBC-iucD*
FMV121/14	2014	Dog	B1	ST539	-	AMP-AMC-CTX-SXT-CIP-NOR	*bla*$_{\text{CTX-M-1type}}$	none	*ecpA*
FMV546/14	2014	Cat	D	ST648	CC648	AMP-AMC-CTX-CAZ-FOX-SXT-CIP-NOR-ENR-CN-TOB	*bla*$_{\text{CMY-2}}$	PAI$_{\text{II536}}$-PAI$_{\text{ICFT073}}$-PAI$_{\text{IV536}}$	*ecpA-papEF*
FMV966/14	2014	Dog	D	ST648	CC648	AMP-AMC-CTX-CAZ-FOX-F-SXT-CIP-NOR-ENR	*bla*$_{\text{CMY-2}}$	II536,ICFT073,IV536	*ecpA-papEF-iucD*
FMV1549/14	2014	Cat	D	ST648	CC648	AMP-AMC-CAZ-FOX-CIP-NOR-ENR	*bla*$_{\text{CMY-2}}$	PAI$_{\text{ICFT073}}$-PAI$_{\text{IV536}}$	*ecpA-papEF*
FMV43/14	2014	Cat	D	ST648	CC648	AMP-AMC-CTX-CAZ-FOX-CIP-NOR-ENR	*bla*$_{\text{CMY-2}}$	PAI$_{\text{ICFT073}}$-PAI$_{\text{IV536}}$	*ecpA-cnf1-sfaDE-papEF*

FMV78/15	2015	Dog	A	ST88	CC23	AMP-CTX-CAZ-SXT-CN-TOB	$bla_{CTX-M-1}$	PAI_{IV536}	*ecpA*
FMV97/15	2015	Dog	B2	O25b:H4-ST131	CC131	AMP-AMC-FOX	bla_{CMY-2}	PAI_{I536}-PAI_{IIJ96}-$PAI_{ICFT073}$-PAI_{IV53}6-$PAI_{IICFT073}$	*ecpA-usp-hlyA-cnf1-sfaDE-papEF-iucD*
FMV4995/15	2015	Dog	B2	ST372	-	AMP-CTX-CAZ	$bla_{CTX-M-15}$	PAI_{IIJ96}-$PAI_{ICFT073}$-PAI_{IV536}-$PAI_{IICFT073}$	*ecpA-hlyA-cnf1-sfaDE-papEF*
FMVCP39/15	2015	Cat	D	ST3258	-	AMP-CTX-SXT	bla_{CMY-2}	$PAI_{ICFT073}$-PAI_{IV536}	*ecpA-iucD*
FMV151/15	2015	Cat	D	ST648	CC648	AMP-AMC-CTX-CAZ-FOX-SXT-CIP-NOR-ENR-CN	bla_{CMY-2}	PAI_{II536}-$PAI_{ICFT073}$-PAI_{IV536}	*ecpA-hlyA-cnf1-sfaDE-papEF-iucD*

Legend: ESBL, Extended-Spectrum β-lactamases; *p*AmpC, *p*AmpC β-lactamases; AMC, Amoxicilin/clavulanate; AMP, Ampicillin; AK, Amikacin; CAZ, Ceftazidime; CIP, Ciprofloxacin; CN, Gentamycin; CTX, Cefotaxime; ENR, Enrofloxacin; FOX, Cefoxitin; F, Nitrofurantoin; NOR, Norfloxacin; SXT, Trimethoprim/sulphamethoxazole; TOB,Tobramycin; [a]antimicrobial susceptibility was accessed according to Clinical and Laboratory Standards Institute (CLSI) guidelines M100-S29;
Pap fimbriae, papEF operon segment; Sfa fimbriae, *sfa*; Afa afimbrial adhesion, *afa*; alpha-hemolysin operon, *hlyA*; Cytotoxic necrotizing factor-1, *cnf*-1; aerobactin siderophore, *iucD*; *E. coli* common pilus, *ecpA;* uropathogenic specific protein, *usp.* [b] New ST allelic profile - Marques et al., 2017.

Table S2: Genotypic characteristics of ESBLs/*p*AmpC-producing *E. coli* strains from humans with UTI (*N*=85) from 2013

strain	Phylogenetic group	Clonal group	CC	Antimicrobial resistance[a]	ESBL and/or *pAmpC* genes	Pathogenicity island markers (PAIs)	Virulence genes
FMVPPL402	A	ST10	CC10	AML-CTX-SXT-CIP-NOR	$bla_{\text{CTX-M-1}}$	PAI$_{\text{IV536}}$	*ecpA-papEF*
FMVML22190	A	ST10	CC10	AML-CTX-SXT-CIP-NOR	$bla_{\text{CTX-M-1}}$	PAI$_{\text{IV536}}$	*ecpA*
FMVPCA14	A	ST88	CC23	AML-CTX-CAZ-SXT-TOB	$bla_{\text{CTX-M-32}}$	PAI$_{\text{IV536}}$	*ecpA*
FMVANZ1442	A	ST88	CC23	AML-AMC-CTX-SXT-CIP-NOR-CN-TOB	$bla_{\text{CTX-M-15}}$	PAI$_{\text{ICFT073}}$-PAI$_{\text{IV536}}$	*ecpA-papEF-iucD*
FMVPLO664	A	ST90	CC23	AML-CTX-SXT-CIP-NOR	$bla_{\text{CTX-M-27}}$	PAI$_{\text{IV536}}$	*ecpA*
FMVANZ1364	A	ST90	CC23	AML-CTX-SXT-CIP-NOR	$bla_{\text{CTX-M-9like}}$	none	*ecpA*
FMV2222	A	ST167	CC10	AML-CTX-CAZ-SXT-CIP-NOR-CN-TOB	$bla_{\text{CTX-M-32}}$	PAI$_{\text{IV536}}$	*ecpA*
FMV1927	A	ST540	-	AML-CTX-CAZ-SXT	$bla_{\text{CTX-M-32}}$	none	*ecpA*
FMVPCA2118	A	ST617	CC10	AML-CTX-CAZ-SXT-CIP-NOR-CN-TOB	$bla_{\text{CTX-M-1}}$	PAI$_{\text{ICFT073}}$-PAI$_{\text{IV536}}$	*ecpA-iucD*
FMVPRV598	A	ST5257	-	AML-CTX	$bla_{\text{CTX-M-32}}$	none	*ecpA*
FMVCde788	A	ST6023	-	AML-CTX-SXT-CIP-NOR	$bla_{\text{CTX-M-14}}$	none	*ecpA*
FMVML17567	B1	ND	-	AML-CTX-SXT	$bla_{\text{CTX-M-2}}$	none	*ecpA*
FMVMLD457	B1	ST58	CC155	AML-CTX-SXT	$bla_{\text{CTX-M-1}}$	PAI$_{\text{ICFT073}}$-PAI$_{\text{IV536}}$	*ecpA-papEF-iucD*
FMVPOOK18	B1	ST58	CC155	AML-CTX-SXT	$bla_{\text{CTX-M-1}}$	PAI$_{\text{IV536}}$	*ecpA-papEF-afaBC,*
FMVBa417	B1	ST453	CC86	AML-CTX-SXT-CIP-NOR	$bla_{\text{CTX-M-14}}$	none	*ecpA*
FMVMQ8509	B1	ST453	CC86	AML-CTX-CAZ-CIP-NOR	$bla_{\text{SHV-12}}$	PAI$_{\text{ICFT073}}$-PAI$_{\text{IV536}}$	*ecpA-iucD*
FMVJP842	B1	ST453	CC86	AML-CTX-SXT-CIP-NOR	$bla_{\text{CTX-M-14}}$	PAI$_{\text{ICFT073}}$-PAI$_{\text{IV536}}$	*ecpA-papEF-iucD*
FMVPRV129e	B1	ST453	CC86	AML-AMC-CTX-SXT-CIP-NOR-CN	$bla_{\text{CTX-M-14}}$	PAI$_{\text{ICFT073}}$-PAI$_{\text{IV536}}$	*ecpA-iucD*
FMVML24561	B1	ST453	CC86	AML-CTX-CIP-NOR	$bla_{\text{CTX-M-1}}$	PAI$_{\text{ICFT073}}$-PAI$_{\text{IV536}}$	*ecpA-iucD*
FMVML35760	B1	ST453	CC86	AML-AMC-CTX-CIP-NOR-CN	$bla_{\text{CTX-M-15}}$	PAI$_{\text{ICFT073}}$-PAI$_{\text{IV536}}$	*ecpA-iucD*
FMV2660	B1	ST847	-	AML-CTX-SXT	$bla_{\text{CTX-M-14}}$	PAI$_{\text{ICFT073}}$-PAI$_{\text{IV536}}$	*ecpA-iucD*
FMVMQ11227	B1	ST1725	-	AML-CTX-SXT-CIP-NOR	$bla_{\text{CTX-M-15}}$	PAI$_{\text{ICFT073}}$-PAI$_{\text{IV536}}$	*ecpA-papEF-iucD*
FMVANG80	B1	ST1196	-	AML-CTX-CAZ-SXT-CIP-NOR	$bla_{\text{CTX-M-1}}$	none	*ecpA*
FMVMR1960	B2	O25b:H4-ST131-H30R1	CC131	AML-CTX-CAZ-SXT-CIP-NOR	$bla_{\text{CTX-M-1}}$	PAI$_{\text{I536}}$-PAI$_{\text{IIJ96-I}}$PAI$_{\text{I536}}$-PAI$_{\text{ICFT073}}$- PAI$_{\text{IV536}}$-PAI$_{\text{IICFT073}}$	*ecpA-papEF-hlyA-cnf1-iucD*

FMVPFB 399	B2	O25b:H4-ST131-H30R1	CC131	AML-AMC-CTX-CAZ-SXT-CIP-NOR	*bla*$_{CTX-M-1}$	PAI$_{ICFT073}$-PAI$_{IV536}$-PAI$_{IICFT073}$	*ecpA-iucD*
FMVOM1969	B2	O25b:H4-ST131-H30Rx	CC131	AML-CTX-CAZ-SXT-CIP-NOR-CN-TOB	*bla*$_{CTX-M-15}$	PAI$_{I536}$-PAI$_{IIJ96}$-PAI$_{II536}$-PAI$_{ICFT073}$-PAI$_{IV536}$-PAI$_{IICFT073}$	*ecpA-papEF-sfaDE-hlyA-cnf1-iucD*
FMVO5N38	B2	O25b:H4-ST131-H30Rx	CC131	AML-CTX-CAZ-SXT-CIP-NOR-TOB	*bla*$_{CTX-M-15}$	PAI$_{I536}$-PAI$_{IIJ96}$-PAI$_{II536}$-PAI$_{ICFT073}$-PAI$_{IV536}$-PAI$_{IICFT073}$	*ecpA-papEF-sfaDE-hlyA-cnf1-iucD*
FMVANG 39	B2	O25b:H4-ST131-H30Rx	CC131	AML-CTX-SXT-CIP-NOR-TOB	*bla*$_{CTX-M-15}$	PAI$_{IIJ96}$-PAI$_{II536}$-PAI$_{ICFT073}$-PAI$_{IV536}$-PAI$_{IICFT073}$	*ecpA-afaBC-hlyA-cnf1-iucD*
FMVMLD 252	B2	O25b:H4-ST131-H30Rx	CC131	AML-AMC-CTX-FOX-SXT-CIP-NOR	*bla*$_{CTX-M-15}$	PAI$_{I536}$-PAI$_{IIJ96}$-PAI$_{IV536}$	*ecpA-hlyA-cnf1*
FMVOMR+80	B2	O25b:H4-ST131-H30Rx	CC131	AML-AMC-CTX-CAZ-SXT-CIP-NOR-CN-TOB-AK	*bla*$_{CTX-M-15}$	PAI$_{ICFT073}$-PAI$_{IV536}$-PAI$_{IICFT073}$	*ecpA-iucD*
FMVNan19	B2	O25b:H4-ST131-H30Rx	CC131	AML-CTX-SXT-CIP-NOR-CN-TOB	*bla*$_{CTX-M-15}$	PAI$_{I536}$-PAI$_{IIJ96}$-PAI$_{II536}$-PAI$_{ICFT073}$-PAI$_{IV536}$-PAI$_{IICFT073}$	*ecpA-papEF-hlyA-cnf1-iucD*
FMVPCV394	B2	O25b:H4-ST131-H30Rx	CC131	AML-CTX-CAZ-CIP-NOR-CN-TOB	*bla*$_{CTX-M-15}$	PAI$_{ICFT073}$-PAI$_{IV536}$-PAI$_{IICFT073}$	*ecpA-iucD*
FMVPAV806	B2	O25b:H4-ST131-H30Rx	CC131	AML-CTX-CAZ-CIP-NOR-CN-TOB	*bla*$_{CTX-M-15}$	PAI$_{I536}$-PAI$_{IIJ96}$-PAI$_{II536}$-PAI$_{ICFT073}$-PAI$_{IV536}$-PAI$_{IICFT073}$	*ecpA-papEF-hlyA-cnf1-iucD*
FMVNA255	B2	O25b:H4-ST131-H30Rx	CC131	AML-AMC-CTX-SXT-CIP-NOR-CN-TOB	*bla*$_{CTX-M-15}$	PAI$_{I536}$-PAI$_{IIJ96}$-PAI$_{II536}$-PAI$_{ICFT073}$PAI-$_{IV536}$-PAI$_{IICFT073}$	*ecpA-papEF-hlyA-cnf1-iucD*
FMVVIM 1771	B2	O25b:H4-ST131-H30Rx	CC131	AML-AMC-CTX-CAZ-F-SXT-CIP-NOR-CN-TOB	*bla*$_{CTX-M-15}$	PAI$_{I536}$-PAI$_{IIJ96}$-PAI$_{II536}$-PAI$_{ICFT073}$-PAI$_{IV536}$-PAI$_{IICFT073}$	*ecpA-papEF-hlyA-cnf1-iucD*
FMVAH6430	B2	O25b:H4-ST131-H30Rx	CC131	AML-AMC-CTX-SXT-CIP-NOR-TOB	*bla*$_{CTX-M-15}$	PAI$_{ICFT073}$-PAI$_{IV536}$-PAI$_{IICFT073}$	*ecpA-papEF- afaBC- iucD*
FMVPME 508	B2	O25b:H4-ST131-H30Rx	CC131	AML-CTX-CAZ-SXT-CIP-NOR-CN-TOB	*bla*$_{CTX-M-15}$	PAI$_{I536}$-PAI$_{IIJ96}$-PAI$_{II536}$-PAI$_{ICFT073}$-PAI $_{IV536}$-PAI $_{IICFT073}$	*ecpA-papEF-sfaDE-hlyA-cnf1-iucD*

FMVPSF1626	B2	O25b:H4-ST131-H30Rx	CC131	AML-CTX-CAZ-SXT-CIP-NOR-TOB	*bla*$_{CTX-M-15}$	PAI$_{I536}$-PAI$_{IIJ96}$-PAI$_{II536}$-PAI$_{ICFT073}$- PAI$_{IV536}$-PAI$_{IICFT073}$	*ecpA-papEF-sfaDE-hlyA-cnf1-iucD*
FMVPSF1301	B2	O25b:H4-ST131-H30Rx	CC131	AML-CTX-CAZ-SXT-CIP-NOR-TOB	*bla*$_{CTX-M-15}$	PAI$_{ICFT073}$-$_{I}$PAI$_{V536}$-PAI$_{IICFT073}$	*ecpA-iucD*
FMVPD26e5	B2	O25b:H4-ST131-H30Rx	CC131	AML-CTX-CIP-NOR	*bla*$_{CTX-M-15}$	PAI$_{I536}$-PAI$_{IV536}$-PAI$_{IICFT073}$	*ecpA-papEF-hlyA-iucD*
FMVML 27331	B2	O25b:H4-ST131-H30Rx	CC131	AML-CTX-SXT-CIP-NOR-CN-TOB	*bla*$_{CTX-M-15}$	PAI$_{I536}$-PAI$_{IIJ96}$-PAI$_{II536}$-PAI$_{ICFT073}$-PAI$_{IV536}$-PAI$_{IICFT073}$	*ecpA-papEF-sfaDE-hlyA-cnf1-iucD*
FMVOMV 168	B2	O25b:H4-ST131-H30Rx	CC131	AML-CTX-CAZ-CIP-NOR-CN-AK	*bla*$_{CTX-M-15}$	PAI$_{ICFT073}$-PAI$_{IV536}$-PAI$_{IICFT073}$	*ecpA-iucD*
PFMVD5379	B2	O25b:H4-ST131-H30Rx	CC131	AML-CTX-SXT-CIP-NOR-CN-TOB	*bla*$_{CTX-M-15}$	PAI$_{I536}$-PAI$_{IIJ96}$-PAI$_{II536}$-PAI$_{ICFT073}$- PAI$_{IV536}$, PAI$_{IICFT073}$	*ecpA-papEF-sfaDE-hlyA-cnf1-iucD*
FMVML25380	B2	O25b:H4-ST131-H30Rx	CC131	AML-CTX-CIP-NOR-CN-TOB	*bla*$_{CTX-M-15}$	PAI$_{I536}$-PAI$_{IIJ96}$-PAI$_{II536}$-PAI$_{ICFT073}$- PAI$_{IV536}$-PAI$_{IICFT073}$	*ecpA-papEF-sfaDE-hlyA-cnf1-iucD*
FMVL5449	B2	O25b:H4-ST131-H30Rx	CC131	AML-CTX-CAZ-SXT-CIP-NOR-TOB	*bla*$_{CTX-M-15}$	PAI$_{I536}$-PAI$_{IIJ96}$-PAI$_{II536}$-PAI$_{ICFT073}$-PAI$_{IV536}$-PAI$_{IICFT073}$	*ecpA-papEF-hlyA-cnf1-iucD*
FMVPD6085	B2	O25b:H4-ST131-H30Rx	CC131	AML-CTX-CIP-NOR-CN-TOB-AK	*bla*$_{CTX-M-15}$	PAI$_{I536}$-PAI$_{IIJ96}$-PAI$_{II536}$-PAI$_{ICFT073}$-PAI$_{IV536}$-PAI$_{IICFT073}$	*ecpA-papEF-hlyA-cnf1-iucD*
FMVANG82	B2	O25b:H4-ST131-H30Rx	CC131	AML-AMC-CTX-CAZ-CIP-NOR-CN-TOB	*bla*$_{CTX-M-15}$	PAI$_{I536}$-PAI$_{IIJ96}$-PAI$_{II536}$-PAI$_{ICFT073}$- PAI$_{IV536}$-PAI$_{IICFT073}$	*ecpA-papEF-sfaDE-hlyA-cnf1-iucD*
FMVJA861	B2	O25b:H4-ST131-H30Rx	CC131	AML-CTX-CAZ-CIP-NOR-TOB	*bla*$_{CTX-M-15}$	PAI$_{I536}$-PAI$_{IIJ96}$-PAI$_{II536}$-PAI$_{ICFT073}$-PAI $_{IV536}$-PAI$_{IICFT073}$	*ecpA-papEF-sfaDE-hlyA-cnf1-iucD*
FMVMR1533	B2	O25b:H4-ST131-H30Rx	CC131	AML-AMC-CTX-SXT-CIP-NOR-CN-TOB	*bla*$_{CTX-M-15}$	PAI$_{I536}$-PAI$_{IIJ96}$-PAI$_{II536}$-PAI$_{ICFT073}$-PAI$_{IV536}$-PAI$_{IICFT073}$	*ecpA-papEF-sfaDE-hlyA-cnf1-iucD*

FMVMQ11181	B2	O25b:H4-ST131-H30Rx	CC131	AML-CTX-SXT-CIP-NOR	*bla*$_{CTX-M-15}$	PAI$_{I536}$-PAI$_{IIJ96}$-PAI$_{II536}$-PAI$_{ICFT073}$-PAI$_{IV536}$-PAI$_{IICFT073}$	*ecpA-papEF-sfaDE-hlyA-cnf1-iucD*
FMVPCV690	B2	O25b:H4-ST131-H30Rx	CC131	AML-CTX-CAZ-CIP-NOR-CN-TOB	*bla*$_{CTX-M-15}$	PAI$_{I536}$-PAI$_{IIJ96}$-PAI$_{II536}$-PAI$_{ICFT073}$-PAI$_{IV536}$-PAI$_{IICFT073}$	*ecpA-papEF-sfaDE-hlyA-cnf1-iucD*
FMVLum380	B2	O25b:H4-ST131-H30Rx	CC131	AML-CTX-CAZ-SXT-CIP-NOR-TOB	*bla*$_{CTX-M-15}$	PAI$_{I536}$-PAI$_{IIJ96}$-PAI$_{II536}$-PAI$_{ICFT073}$-PAI$_{IV536}$-PAI$_{IICFT073}$	*ecpA-papEF-sfaDE-hlyA-cnf1-iucD*
FMVCT313	B2	O25b:H4-ST131-H30Rx	CC131	AML-CTX-CAZ-SXT-CIP-NOR-CN-TOB	*bla*$_{CTX-M-15}$	PAI$_{ICFT073}$-PAI$_{IV536}$-PAI$_{IICFT073}$	*ecpA-iucD*
FMVML28567	B2	O25b:H4-ST131-H30Rx	CC131	AML-AMC-CTX-CAZ-SXT-CIP-NOR-TOB-AK	*bla*$_{CTX-M-15}$	PAI$_{ICFT073}$-PAI$_{IV536}$-PAI$_{IICFT073}$	*ecpA-afaBC-iucD*
FMVMQeeo5	B2	O25b:H4-ST131-H30Rx	CC131	AML-CTX-SXT-CIP-NOR-CN-TOB	*bla*$_{CTX-M-15}$	PAI$_{ICFT073}$-PAI$_{IV536}$-PAI$_{IICFT073}$	*ecpA-iucD*
FMVML34063	B2	O25b:H4-ST131-H30Rx	CC131	AML-CTX-SXT-CIP-NOR-CN-TOB	*bla*$_{CTX-M-15}$	PAI$_{I536}$-PAI$_{IIJ96}$-PAI$_{II536}$-PAI$_{ICFT073}$- PAI$_{IV536}$-PAI$_{IICFT073}$	*ecpA-papEF-hlyA-cnf1-iucD*
FMVAM1035	B2	O25b:H4-ST131-H30Rx	CC131	AML-AMC-CTX-SXT-CIP-NOR-TOB-AK	*bla*$_{CTX-M-15}$	PAI$_{ICFT073}$-PAI$_{IV536}$-PAI$_{IICFT073}$	*ecpA-sfaDE-iucD*
FMVOSF729	B2	O25b:H4-ST131-H30Rx	CC131	AML-AMC-CTX-CAZ-CIP-NOR-CN-TOB-AK	*bla*$_{CTX-M-15}$	PAI$_{ICFT073}$-PAI$_{IV536}$-PAI$_{IICFT073}$	*ecpA-iucD*
FMVMQX1101	B2	O25b:H4-ST131-H30Rx	CC131	AML-AMC-CTX-CAZ-CIP-NOR-CN	*bla*$_{CTX-M-15}$	PAI$_{I536}$-PAI$_{IIJ96}$-PAI$_{II536}$-PAI$_{IV536}$-PAI$_{IICFT073}$	*ecpA-hlyA-cnf1-iucD*
FMVRR3043	B2	O25b:H4-ST131-H30Rx	CC131	AML-CTX-CAZ-SXT-CIP-NOR-CN-TOB-AK	*bla*$_{CTX-M-15}$	PAI$_{ICFT073}$-PAI$_{IV536}$-PAI$_{IICFT073}$	*ecpA-iucD*
FMVPcue031	B2	O25b:H4-ST131-H30Rx	CC131	AML-CTX-CAZ-CIP-NOR-CN-TOB-AK	*bla*$_{CTX-M-15}$	PAI$_{ICFT073}$-PAI$_{IV536}$-PAI$_{IICFT073}$	*ecpA-papEF-iucD*
FMVML36906	B2	O25b:H4-ST131-H30Rx	CC131	AML-CTX-CAZ-CIP-NOR-TOB-AK	*bla*$_{CTX-M-15}$	PAI$_{ICFT073}$-PAI$_{IV536}$-PAI$_{IICFT073}$	*ecpA-iucD*
FMVPME856	B2	O25b:H4-ST131-H30Rx	CC131	AML-CXT-SXT-CIP-NOR-CN-AK	*bla*$_{CTX-M-15}$	PAI$_{I536}$-PAI$_{IIJ96}$-PAI$_{II536}$-PAI$_{ICFT073}$- PAI$_{IV536}$-PAI$_{IICFT073}$	*ecpA-papEF-sfaDE-hlyA-cnf1-iucD*

FMVCS4641	B2	O25b:H4-ST131-H30Rx	CC131	AML-CTX-SXT-CIP-NOR-TOB	*bla*$_{CTX-M-15}$	PAI$_{I536}$-PAI$_{IIJ96}$-PAI$_{II536}$-PAI$_{ICFT073}$-PAI $_{IV536}$-PAI$_{IICFT073}$	*ecpA-papEF-hlyA-cnf1-iucD*
FMVCS4642	B2	O25b:H4-ST131-H30Rx	CC131	AML-CTX-SXT-CIP-NOR-TOB	*bla*$_{CTX-M-15}$	PAI$_{ICFT073}$-PAI$_{IV536}$-PAI$_{IICFT073}$	*ecpA-afaBC-iucD*
FMVLi8696	B2	O25b:H4-ST131-H30Rx	CC131	AML-CTX-SXT-CIP-NOR-TOB	*bla*$_{CTX-M-15}$	PAI$_{ICFT073}$-PAI$_{IV536}$-PAI$_{IICFT073}$	*ecpA-papEF-afaBC-iucD*
FMVAFE 711	B2	O25b:H4-ST131	CC131	AML-AMC-CTX-CAZ-CIP-NOR-CN-TOB	*bla*$_{CTX-M-15}$	PAI$_{ICFT073}$-PAI$_{IV536}$-PAI$_{IICFT073}$	*ecpA-iucD*
FMVPCA456	B2	O25b:H4-ST131-H30R1	CC131	AML-CTX-CAZ-CIP-NOR	*bla*$_{CTX-M-32}$	PAI$_{I536}$-PAI$_{IIJ96}$-PAI$_{II536}$-PAI$_{ICFT073}$- PAI$_{IV536}$-PAI$_{IICFT073}$	*ecpA-papEF-hlyA-cnf1-iucD*
FMVLF4809	B2	O16:H5-ST131	CC131	AML-CTX-SXT-CIP-NOR-CN-TOB	*bla*$_{CTX-M-9like}$	PAI$_{ICFT073}$-PAI$_{IV536}$-PAI$_{IICFT073}$	*ecpA-iucD*
FMVUF822	B2	O25b:H4-ST131-H30Rx	CC131	AML-CXT-SXT-CIP-NOR-CN	*bla*$_{CTX-M-9like}$	PAI$_{I536}$-PAI$_{IIJ96-I}$PAI$_{I536}$-PAI$_{ICFT073}$- PAI$_{IV536}$-PAI$_{IICFT073}$	*ecpA-papEF-hlyA-cnf1-iucD*
FMVML36741	B2	025b:H4-ST131	CC131	AML-AMC-CTX	*bla*$_{CTX-M-9like}$	PAI$_{ICFT073}$-PAI$_{IV536}$-PAI$_{IICFT073}$	*ecpA-iucD*
FMVNAL 47	B2	O25b:H4-ST131-H30R1	CC131	AML-AMC-CTX-FOX-SXT-CIP-NOR	*bla*$_{CTX-M-14}$	PAI$_{ICFT073}$-PAI$_{IV536}$-PAI$_{IICFT073}$	*ecpA-iucD*
FMVPF8675	B2	O25b:H4-ST131-H30R1	CC131	AML-CTX-SXT-CIP-NOR	*bla*$_{CTX-M-27}$	PAI$_{ICFT073}$-PAI$_{IV536}$-PAI$_{IICFT073}$	*ecpA-iucD*
FMVOSJ589	B2	O25b:H4-ST131-H30R1	CC131	AML-CTX-SXT-CIP-NOR	*bla*$_{CTX-M-27}$	PAI$_{I536}$-PAI$_{IIJ96}$-PAI$_{ICFT073}$-PAI$_{IV536}$-PAI$_{IICFT073}$	*ecpA-papEF-sfaDE-hlyA-cnf1-iucD*
FMVCD3e67	B2	O25b:H4-ST131-H30R1	CC131	AML-CTX-SXT-CIP-NOR	*bla*$_{CTX-M-27}$	PAI$_{I536}$-PAI$_{IIJ96}$-PAI$_{ICFT073}$-PAI$_{IV536}$-PAI$_{IICFT073}$	*ecpA-hlyA-cnf1-iucD*
FMVANZ1648	B2	O25b:H4-ST131-H30R1	CC131	AML-CTX-SXT-CIP-NOR	*bla*$_{CTX-M-27}$	PAI$_{ICFT073}$-PAI$_{IV536}$-PAI$_{IICFT073}$	*ecpA-iucD*
FMVOSJ600	B2	O25b:H4-ST131-H30R1	CC131	AML-CTX-SXT-CIP-NOR	*bla*$_{CTX-M-27}$	PAI$_{I536}$-PAI$_{IIJ96}$-PAI$_{ICFT073}$-PAI$_{IV536}$-PAI$_{IICFT073}$	*ecpA-hlyA-cnf1-iucD*

FMVRO757	B2	O25b:H4-ST131	CC131	AML-AMC-CTX-CAZ-FOX-SXT	*bla*$_{CMY-2}$	PAI$_{I536}$-PAI$_{IIJ96}$-PAI$_{II536}$-PAI$_{ICFT073}$- PAI$_{IV536}$-PAI$_{IICFT073}$	*ecpA-papEF-hlyA-cnf1-iucD*
FMVPSF1269	B2	O25b:H4-ST131	CC131	AML-AMC-CTX-FOX-F-SXT-CIP-NOR	*bla*$_{CMY-2}$	PAI$_{ICFT073}$-PAI$_{IV536}$-PAI$_{IICFT073}$	*ecpA-iucD*
FMVRR2968	B2	O25b:H4-ST131	CC131	AML-AMC-CTX-CAZ-FOX-CIP-NOR	*bla*$_{CMY-2}$	PAI$_{I536}$-PAI$_{IIJ96}$-PAI$_{II536}$-PAI$_{ICFT073}$- $_{IV536}$- $_{IICFT073}$	*ecpA-papEF-hlyA-cnf1-iucD*
FMVMQ13896	D	ST117	-	AML-CTX-CAZ-CIP-NOR	*bla*$_{CTX-M-15}$	PAI$_{IIJ96}$-PAI$_{IV536}$-PAI$_{IICFT073}$	*ecpA-papEF-hlyA-cnf1-iucD*
FMVUF628	D	ST354	CC354	AML-CTX-SXT_CIP-NOR-CN	*bla*$_{CTX-M-14}$	PAI$_{ICFT073}$-PAI$_{IV536}$-PAI$_{IICFT073}$	*ecpA-iucD*
FMVMC2815	D	ST410	CC23	AML-CTX-SXT-CIP-NOR	*bla*$_{CTX-M-15}$	PAI$_{I536}$-PAI$_{IIJ96}$-PAI$_{II536}$-PAI$_{IV536}$	*ecpA-papEF-sfaDE-afaBC-hlyA-cnf1-iucD*
FMVLi3864	D	ST648	CC648	AML-AMC-CTX-CAZ-FOX-SXT-CIP-NOR	*bla*$_{CMY-2}$	PAI$_{ICFT073}$-PAI$_{IV536}$	*ecpA- iucD*
FMVPD4783	D	ST778	CC38	AML-AMC-CTX-CAZ-FOX-SXT	*bla*$_{CMY-2}$	PAI$_{ICFT073}$-PAI$_{IV536}$	*ecpA-papEF-iucD*

Legend: ESBL, Extended-Spectrum β-lactamases; *p*AmpC, *p*AmpC β-lactamases; AMC, Amoxicilin/clavulanate; AML, Amoxicilin; AK, Amikacin; CAZ, Ceftazidime; CIP, Ciprofloxacin; CN, Gentamycin; CTX, Cefotaxime; ENR, Enrofloxacin; FOX, Cefoxitin; F, Nitrofurantoin; NOR, Norfloxacin; SXT, Trimethoprim/sulphamethoxazole; TOB,Tobramycin; [a]antimicrobial susceptibility was accessed according to Clinical and Laboratory Standards Institute (CLSI) guidelines M100-S29; Pap fimbriae, papEF operon segment; Sfa fimbriae, *sfa*; Afa afimbrial adhesion, *afa*; alpha-hemolysin operon, *hlyA*; Cytotoxic necrotizing factor-1, *cnf*-1; aerobactin siderophore, *iucD*; *E. coli* common pilus, *ecpA;* uropathogenic specific protein, *usp.*
